# Molecular Mechanisms Underlying the Cellular Entry and Host Range Restriction of Lujo Virus

**DOI:** 10.1128/mbio.03060-21

**Published:** 2022-02-15

**Authors:** Takeshi Saito, Takanari Hattori, Kosuke Okuya, Rashid Manzoor, Hiroko Miyamoto, Masahiro Kajihara, Ayato Takada

**Affiliations:** a Division of Global Epidemiology, International Institute for Zoonosis Control, Hokkaido University, Sapporo, Japan; b International Collaboration Unit, International Institute for Zoonosis Control, Hokkaido University, Sapporo, Japan; c Department of Disease Control, School of Veterinary Medicine, University of Zambia, Lusaka, Zambia; Icahn School of Medicine at Mount Sinai

**Keywords:** arenavirus, CD63, host range, LUJV, Lujo virus, membrane fusion, viral entry, glycoprotein

## Abstract

Like other human-pathogenic arenaviruses, Lujo virus (LUJV) is a causative agent of viral hemorrhagic fever in humans. LUJV infects humans with high mortality rates, but the susceptibilities of other animal species and the molecular determinants of its host specificity remain unknown. We found that mouse- and hamster-derived cell lines (NIH 3T3 and BHK, respectively) were less susceptible to a replication-incompetent recombinant vesicular stomatitis virus (Indiana) pseudotyped with the LUJV glycoprotein (GP) (VSVΔG*-LUJV/GP) than were human-derived cell lines (HEK293T and Huh7). To determine the cellular factors involved in the differential susceptibilities between the human and mouse cell lines, we focused on the CD63 molecule, which is required for pH-activated GP-mediated membrane fusion during LUJV entry into host cells. The exogenous introduction of human CD63, but not mouse or hamster CD63, into BHK cells significantly increased susceptibility to VSVΔG*-LUJV/GP. Using chimeric human-mouse CD63 proteins, we found that the amino acid residues at positions 141 to 150 in the large extracellular loop (LEL) region of CD63 were important for the cellular entry of VSVΔG*-LUJV/GP. By site-directed mutagenesis, we further determined that a phenylalanine at position 143 in human CD63 was the key residue for efficient membrane fusion and VSVΔG*-LUJV/GP infection. Our data suggest that the interaction of LUJV GP with the LEL region of CD63 is essential for cell susceptibility to LUJV, thus providing new insights into the molecular mechanisms underlying the cellular entry of LUJV and the host range restriction of this virus.

## INTRODUCTION

Viruses in the family *Arenaviridae* are divided into four genera: *Antennavirus*, *Hartmanivirus*, *Reptarenavirus*, and *Mammarenavirus*. The genus *Mammarenavirus* is classified into two major groups, the Old World (OW) and the New World (NW) arenaviruses, based on their serological, genetic, and geographic relationships. Some mammarenaviruses infect humans and cause diseases ranging from asymptomatic to severe hemorrhagic fever. Of these, Lassa virus (LASV) and Lujo virus (LUJV), OW arenaviruses, are etiological agents of fatal hemorrhagic fevers in humans. NW arenaviruses, including Junin, Machupo, Sabia, Guanarito, and Chapare viruses, are also known to cause hemorrhagic fevers in humans (1, 2). LUJV caused an outbreak in 2008 in Zambia and South Africa. Although the number of patients was limited, the mortality rate reached 80% (1, 3). In general, rodents are natural reservoirs of mammarenaviruses, except for Tacaribe virus isolated from an artibeus bat (2, 4). A natal multimammate mouse (Mastomys natalensis) is known to be the natural host of LASV; however, the reservoirs of LUJV are still unknown (5, 6). To establish effective countermeasures against arenavirus infections, it is important to understand the molecular basis of their pathogenicities and host ranges.

Arenaviruses are enveloped, two-segmented, single-stranded, ambisense RNA viruses. The S segment of the viral genome encodes the glycoprotein (GP) precursor and the nucleoprotein, and the L segment encodes the matrix protein (Z) and the RNA-dependent RNA polymerase (L) (2, 7). GP is synthesized as a single polypeptide chain and cleaved posttranslationally into GP1 and GP2 by a cellular proprotein convertase, subtilisin kexin isozyme 1/site 1 protease (8, 9). GP1 is located on the top of the viral surface GP spike and mediates the attachment of virions to the target cell. GP2 contains the transmembrane region of the GP spike and mediates membrane fusion between the viral envelope and the host cell membrane (7, 10). In accordance with the phylogenetic differences in GP sequences, arenaviruses utilize several different cellular receptors. It is known that mammarenaviruses generally use human transferrin receptor 1 (TfR1) and α-dystroglycan (α-DG) (7, 10). On the other hand, neuropilin-2 (NRP2) and CD63 have been reported to be the cellular factors required for LUJV entry into cells (11). NRP2 acts as the attachment receptor, and the direct interaction between LUJV GP1 and NRP2 has been well investigated (11, 12). CD63 is thought to be required for pH-activated GP-mediated membrane fusion in endosomes. However, the detailed mechanisms by which the interaction between CD63 and LUJV GP triggers membrane fusion are still unclear (11).

Although LUJV causes severe and life-threatening hemorrhagic fevers in humans, previous reports suggested different susceptibilities to LUJV infection among animal species. Cynomolgus macaques experimentally infected with LUJV displayed only mild, nonlethal illness (13). More prominent differences in susceptibility to LUJV were reported among rodents. LUJV did not cause any clinical symptoms or mortality in newborn and weanling mice, whereas LUJV-infected guinea pigs developed hemorrhagic manifestations similar to the symptoms observed in human cases (14). However, neither viral nor cellular factors that explain such differential susceptibilities among animal species have been elucidated.

To determine the molecular mechanisms underlying the potential host specificity of LUJV, we focused on the difference between human and rodent CD63 proteins. Using a replication-incompetent vesicular stomatitis virus (VSV) pseudotyped with LUJV GP (15), we found that rodent-derived cell lines showed significantly lower susceptibility than the human-derived cell lines tested and further demonstrated that a single amino acid residue located in the large extracellular loop (LEL) of CD63 was essential for the differential susceptibility to the virus. Our results suggest the importance of the interaction of LUJV GP with the LEL region of CD63 for LUJV infection and also provide useful information for understanding the molecular basis of the host range of LUJV.

## RESULTS

### Differential susceptibilities of human- and rodent-derived cell lines to VSV pseudotyped with LUJV GP and effects of NRP2 and CD63 expression on the susceptibility of BHK cells.

**FIG 1:**
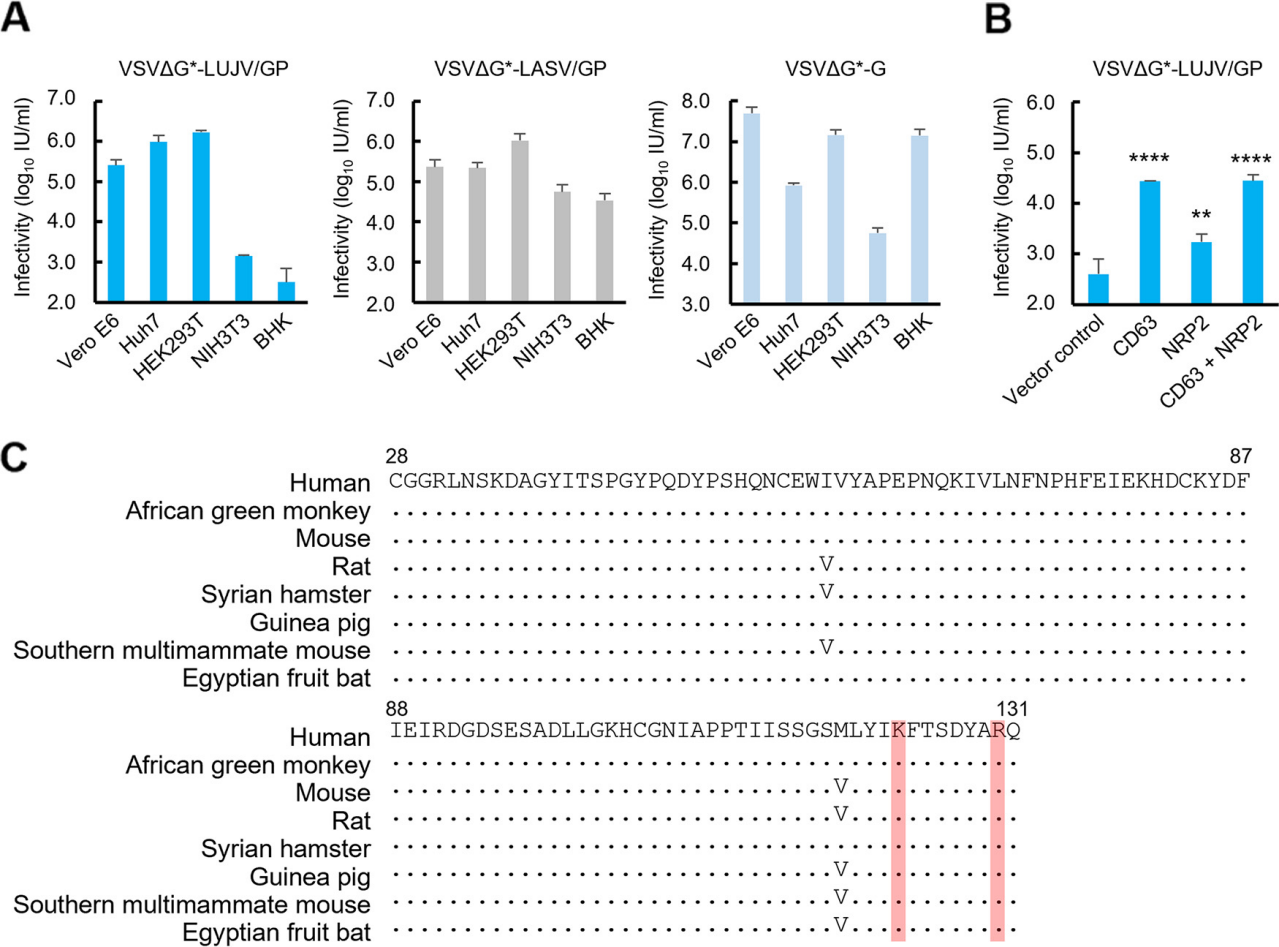
Differential susceptibilities of primate- and rodent-derived cell lines to pseudotyped VSVs and importance of CD63 for LUJV entry into cells. (A) Infectious units (IU) of VSVΔG*-LUJV/GP, VSVΔG*-LASV/GP, and VSVΔG*-G were determined in Vero E6, Huh7, HEK293T, NIH 3T3, and BHK cells. Each experiment was conducted three times, and averages and standard deviations are shown. (B) BHK cells transduced with exogenous human CD63 and/or NRP2 genes were infected with VSVΔG*-LUJV/GP. The experiment was conducted three times, and averages and standard deviations are shown. Significant differences compared to the cells transduced with the vector control are shown (*, *P < *0.05; **, *P < *0.01; ***, *P < *0.001; ****, *P < *0.0001). (C) Amino acid sequences of the CUB1 region of NRP2 orthologs are aligned for the human (Homo sapiens [GenBank accession number NP_003863.2]), African green monkey (Chlorocebus sabaeus [accession number XP_007964144.1]), mouse (Mus musculus [accession number NP_001070872.1]), rat (Rattus norvegicus [accession number XP_006245101.1]), Syrian hamster (Mesocricetus auratus [accession number XP_005070706.1]), guinea pig (Cavia porcellus [accession number XP_013012991.1]), Southern multimammate mouse (Mastomys coucha [accession number XP_031223558.1]), and Egyptian fruit bat (Rousettus aegyptiacus [accession number XP_015990573.1]), in human NRP2 numbering. The amino acid residues directly interacting with LUJV GP1 (lysine and arginine at positions 123 and 130, respectively) are highlighted in pale red.

**FIG 2:**
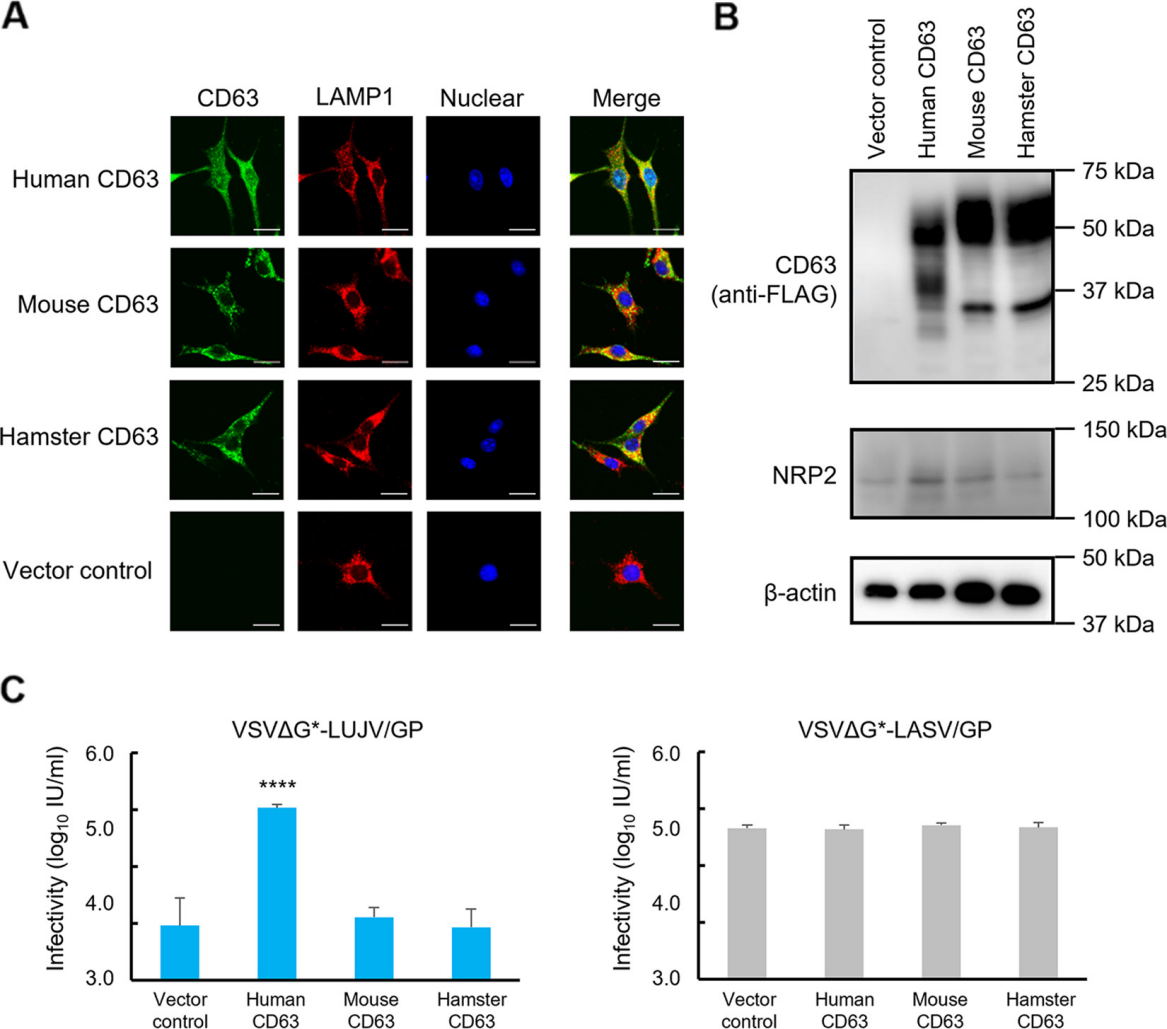
Expression of exogenous human, mouse, and hamster CD63s in BHK cells and infectivities of VSVΔG*-LUJV/GP and VSVΔG*-LASV/GP. (A) The intracellular localization of exogenous CD63s in BHK cells was analyzed by confocal microscopy. Bars, 20 μm. (B) Each cell lysate was separated by SDS-PAGE followed by Western blotting. (C) Exogenous human, mouse, or hamster CD63-expressing BHK cells were infected with VSVΔG*-LUJV/GP or VSVΔG*-LASV/GP, and infectious units (IU) were determined. Each experiment was conducted three times, and averages and standard deviations are shown. Significant differences compared to the cells transduced with the vector control are shown (*, *P < *0.05; **, *P < *0.01; ***, *P < *0.001; ****, *P < *0.0001).

To analyze the susceptibilities of human and rodent cell lines to arenaviruses, Vero E6 (African green monkey kidney), Huh7 (human liver), HEK293T (human kidney), NIH 3T3 (mouse fibroblast), and BHK (hamster kidney) cells were infected with VSV containing the green fluorescent protein (GFP) gene instead of the VSV glycoprotein (G) gene and bearing Lujo virus GP (VSVΔG*-LUJV/GP), Lassa virus GP (VSVΔG*-LASV/GP), or VSV G protein (VSVΔG*-G) (Fig. 1A). We found that unlike the tropism of VSVΔG*-G, VSVΔG*-LUJV/GP and VSVΔG*-LASV/GP infected Vero E6, Huh7, and HEK293T cells more efficiently than NIH 3T3 and BHK cells, and this tendency was particularly prominent for VSVΔG*-LUJV/GP infectivity. Interestingly, VSVΔG*-LUJV/GP showed much lower infectious units (IU) (2 to 3 logs lower) in NIH 3T3 and BHK cells than VSVΔG*-LASV/GP. Notably, BHK cells were almost nonpermissive to VSVΔG*-LUJV/GP. We then assumed that the differential susceptibility was due to the structural differences in the LUJV receptor molecules (i.e., NRP2 and CD63). To confirm the roles of these receptor molecules, we generated BHK cell lines stably expressing exogenous human CD63 and/or NRP2 and examined their susceptibilities to VSVΔG*-LUJV/GP (Fig. 1B). As expected, the expression of these molecules in BHK cells significantly enhanced the infectivity of VSVΔG*-LUJV/GP, whereas only a marginal enhancement was observed for the expression of NRP2. We also generated NIH 3T3 cell lines stably expressing exogenous human CD63 and/or NRP2 to examine their susceptibilities to VSVΔG*-LUJV/GP and confirmed that the expression of CD63 showed much greater effects than NRP2 (see Fig. S1 in the supplemental material). Accordingly, the amino acid sequences of the N-terminal CUB1 domain of NRP2, which is critical for binding between NRP2 and LUJV GP1 (11, 12), are well conserved among mammalian species, and there are no amino acid differences that could explain the lower susceptibility of the rodent cell lines tested, suggesting a limited role in the differential susceptibilities between the human and rodent cell lines (Fig. 1C). We then focused on the CD63 molecule, and BHK cell lines expressing human, mouse, and hamster CD63s were generated for comparison of their susceptibilities to VSVΔG*-LUJV/GP and VSVΔG*-LASV/GP. We confirmed similar intracellular localizations and expression levels of the exogenous CD63s by confocal microscopy and Western blotting (Fig. 2A and B). In contrast to VSVΔG*-LASV/GP, which uniformly infected all these cell lines, VSVΔG*-LUJV/GP showed significantly higher infectivity in human CD63-expressing cells than in the cells expressing mouse or hamster CD63 (Fig. 2C). Similar results were obtained when NIH 3T3 cells were used for the expression of the CD63 molecules (Fig. S2).

10.1128/mBio.03060-21.1 FIG S1 Importance of human CD63 expression for LUJV entry into mouse-derived cells (NIH 3T3) (related to Fig. 1). NIH 3T3 cells transduced with exogenous human CD63 and/or NRP2 genes were infected with VSVΔG*-LUJV/GP and VSVΔG*-G, and infectious units (IU) were determined. Each experiment was conducted three times, and averages and standard deviations are shown. Significant differences compared to the cells transduced with the vector control (Vector control) are shown (*, *P < *0.05; **, *P < *0.01; ***, *P < *0.001; ****, *P < *0.0001). Download FIG S1, PDF file, 0.2 MB. Copyright © 2022 Saito et al. 2022 Saito et al. https://creativecommons.org/licenses/by/4.0/ This content is distributed under the terms of the Creative Commons Attribution 4.0 International license.

10.1128/mBio.03060-21.2 FIG S2 Effects of exogenous human, mouse, and hamster CD63 expression on infectivities of VSVΔG*-LUJV/GP and VSVΔG*-LASV/GP in NIH 3T3 cells (related to Fig. 2). Exogenous human, mouse, or hamster CD63-expressing NIH 3T3 cells were infected with VSVΔG*-LUJV/GP or VSVΔG*-LASV/GP, and infectious units (IU) were determined. Each experiment was conducted three times, and averages and standard deviations are shown. Significant differences compared to the cells transduced with the vector control (Vector control) are shown (*, *P < *0.05; **, *P < *0.01; ***, *P < *0.001; ****, *P < *0.0001). Download FIG S2, PDF file, 0.2 MB. Copyright © 2022 Saito et al. 2022 Saito et al. https://creativecommons.org/licenses/by/4.0/ This content is distributed under the terms of the Creative Commons Attribution 4.0 International license.

### Mapping of the functional regions in the CD63 molecule for VSVΔG*-LUJV/GP infectivity.

**FIG 3:**
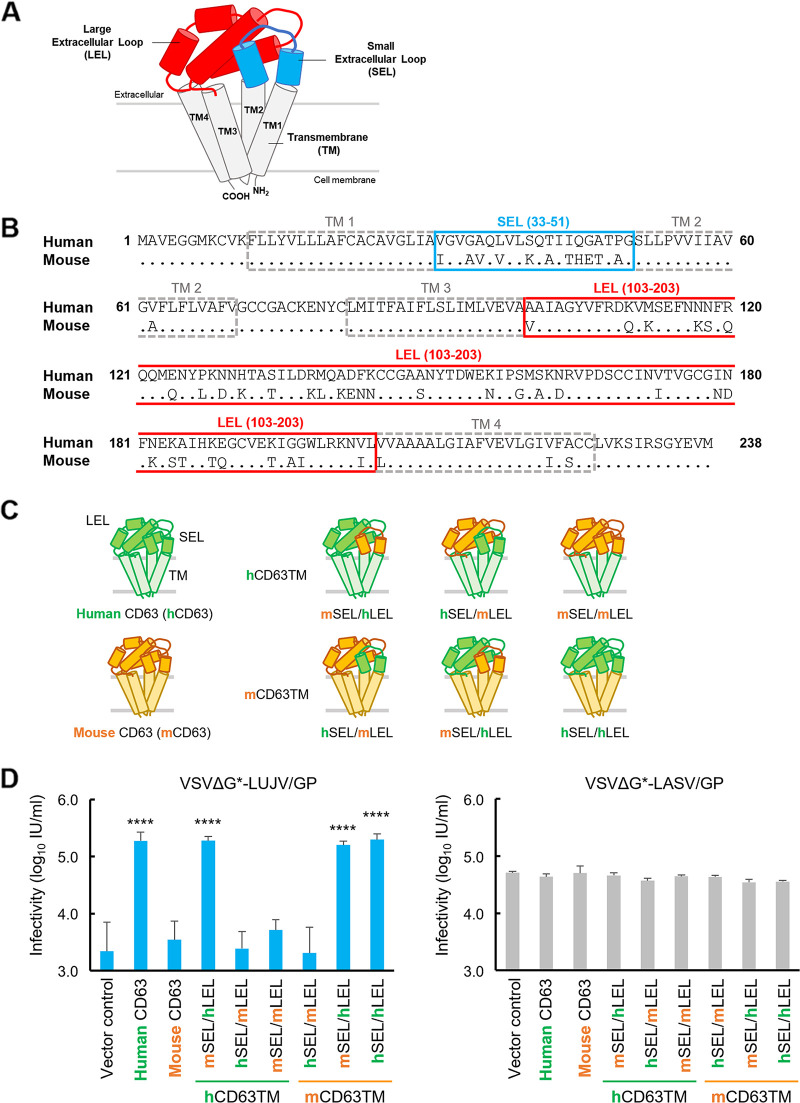
Importance of the large extracellular loop region of human CD63 for VSVΔG*-LUJV/GP entry. (A) Schematic diagram of CD63. CD63 contains four transmembrane helices (TM1 to TM4), an N-terminal small extracellular loop, and a C-terminal large extracellular loop (16). (B) Comparison of amino acid sequences of human CD63 (GenBank accession number NP_001244318.1) and mouse CD63 (accession number NP_001036045.1). (C) Schematic diagram of chimeric CD63s produced in this study. Regions shown in green and orange indicate those derived from human and mouse CD63s, respectively. These chimeric CD63 proteins contain the human CD63 transmembrane domain (hCD63TM) or the mouse CD63 transmembrane domain (mCD63TM). (D) Infectious units (IU) of VSVΔG*-LUJV/GP or VSVΔG*-LASV/G in BHK cells expressing wild-type human, mouse, and chimeric CD63s. Each experiment was conducted three times, and averages and standard deviations are shown. Significant differences compared to the cells transduced with the vector control are shown (*, *P < *0.05; **, *P < *0.01; ***, *P < *0.001; ****, *P < *0.0001).

CD63 is a member of the tetraspanin family, which contains transmembrane (TM) domains and N-terminal small extracellular loop (SEL) and C-terminal large extracellular loop (LEL) regions (16) (Fig. 3A). Our comparative sequence analysis of human and mouse CD63s revealed major differences located in the SEL and LEL regions (Fig. 3B). To investigate which region of human CD63 is important for VSVΔG*-LUJV/GP infection, we generated BHK cell lines stably expressing chimeric human-mouse CD63 proteins and compared their susceptibilities to the viruses (Fig. 3C and D). The expression and intracellular localization of the chimeric CD63s were confirmed by Western blotting and confocal microscopy (Fig. S3). Consistent with the results shown in Fig. 2, all of the cell lines expressing human, mouse, or chimeric CD63 showed similar susceptibilities to VSVΔG*-LASV/GP. In contrast, VSVΔG*-LUJV/GP efficiently infected the cells expressing CD63 containing the human CD63-derived LEL but not the mouse CD63-derived LEL. These data indicated that the LEL region of human CD63 determined the high susceptibility of the human cell lines to VSVΔG*-LUJV/GP.

10.1128/mBio.03060-21.3 FIG S3 Expression of exogenous human and mouse CD63s and their chimeric mutants in BHK cells (related to Fig. 3). (A) The intracellular localization of exogenous chimeric CD63 in BHK cells was analyzed by confocal microscopy as described in Materials and Methods. Bars, 20 μm. (B) Each cell lysate was separated by SDS-PAGE followed by Western blotting as described in Materials and Methods. Download FIG S3, PDF file, 0.7 MB. Copyright © 2022 Saito et al. 2022 Saito et al. https://creativecommons.org/licenses/by/4.0/ This content is distributed under the terms of the Creative Commons Attribution 4.0 International license.

### Contribution of amino acid residues at positions 141 to 150 in CD63 to VSVΔG*-LUJV/GP infection.

**FIG 4:**
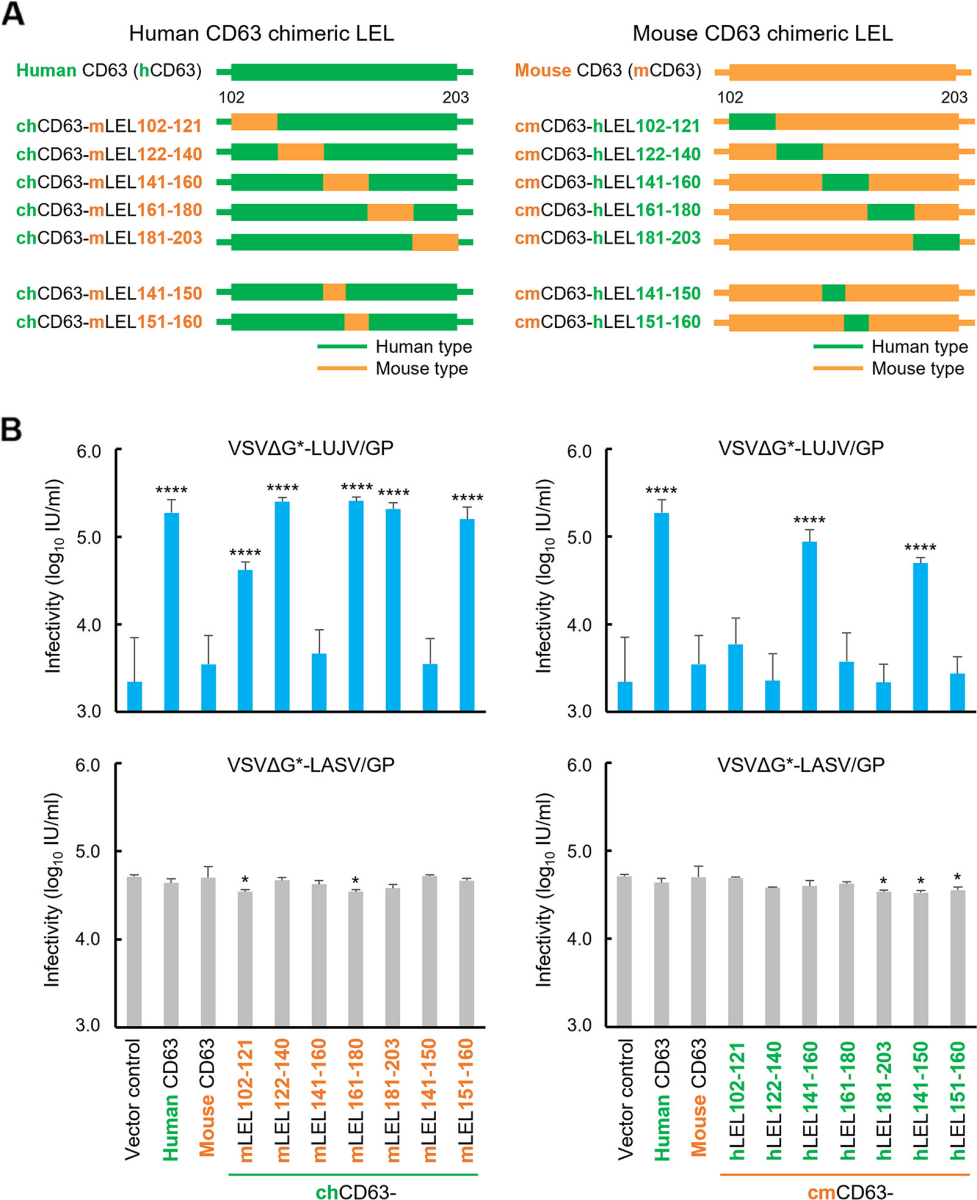
Importance of amino acid positions 141 to 160 of human CD63 for VSVΔG*-LUJV/GP entry. (A) Schematic diagrams of chimeric human and mouse CD63s. Regions shown in green and orange indicate those derived from human and mouse CD63s, respectively. (B) Infectious units (IU) of VSVΔG*-LUJV/GP or VSVΔG*-LASV/GP in BHK cells expressing wild-type human, mouse, and chimeric CD63s. Each experiment was conducted three times, and averages and standard deviations are shown. Significant differences compared to the cells transduced with the vector control are shown (*, *P < *0.05; **, *P < *0.01; ***, *P < *0.001; ****, *P < *0.0001).

To further determine the region important for VSVΔG*-LUJV/GP infection, we tested CD63 mutants having chimeric human-mouse CD63 LEL regions (Fig. 4A). BHK cell lines stably expressing human and mouse wild-type CD63s and the CD63 mutants were infected with VSVΔG*-LUJV/GP and VSVΔG*-LASV/GP to compare their susceptibilities to the viruses (Fig. 4B). The protein expression and intracellular localization were confirmed as described above (Fig. S4). As expected, the expression of chimeric CD63s did not affect the susceptibility to VSVΔG*-LASV/GP. In contrast, human CD63 having amino acid residues from mouse CD63 at positions 141 to 160 (chCD63-mLEL141–160) significantly decreased the ability to mediate VSVΔG*-LUJV/GP entry, and the replacement of this region in mouse CD63 with that of the human LEL (cmCD63-hLEL141–160) significantly increased the susceptibility of the cells. We further generated cells expressing chimeric CD63 in which the amino acid residues at positions 141 to 150 and 151 to 160 were swapped (chCD63-mLEL141–150, chCD63-mLEL151–160, cmCD63-hLEL141–150, and cmCD63-hLEL151–160) and found that the amino acid residues at positions 141 to 150 of human CD63 were critical for VSVΔG*-LUJV/GP infection (Fig. 4B).

10.1128/mBio.03060-21.4 FIG S4 Expression of exogenous human and mouse CD63s and their mutants having the chimeric LEL regions in BHK cells (related to Fig. 4). (A) The intracellular localization of CD63 in BHK cells was analyzed by confocal microscopy as described in Materials and Methods. Bars, 20 μm. (B) Each cell lysate was separated by SDS-PAGE followed by Western blotting as described in Materials and Methods. Download FIG S4, PDF file, 0.7 MB. Copyright © 2022 Saito et al. 2022 Saito et al. https://creativecommons.org/licenses/by/4.0/ This content is distributed under the terms of the Creative Commons Attribution 4.0 International license.

### Importance of the phenylalanine at position 143 in human CD63 for VSVΔG*-LUJV/GP infection.

**FIG 5:**
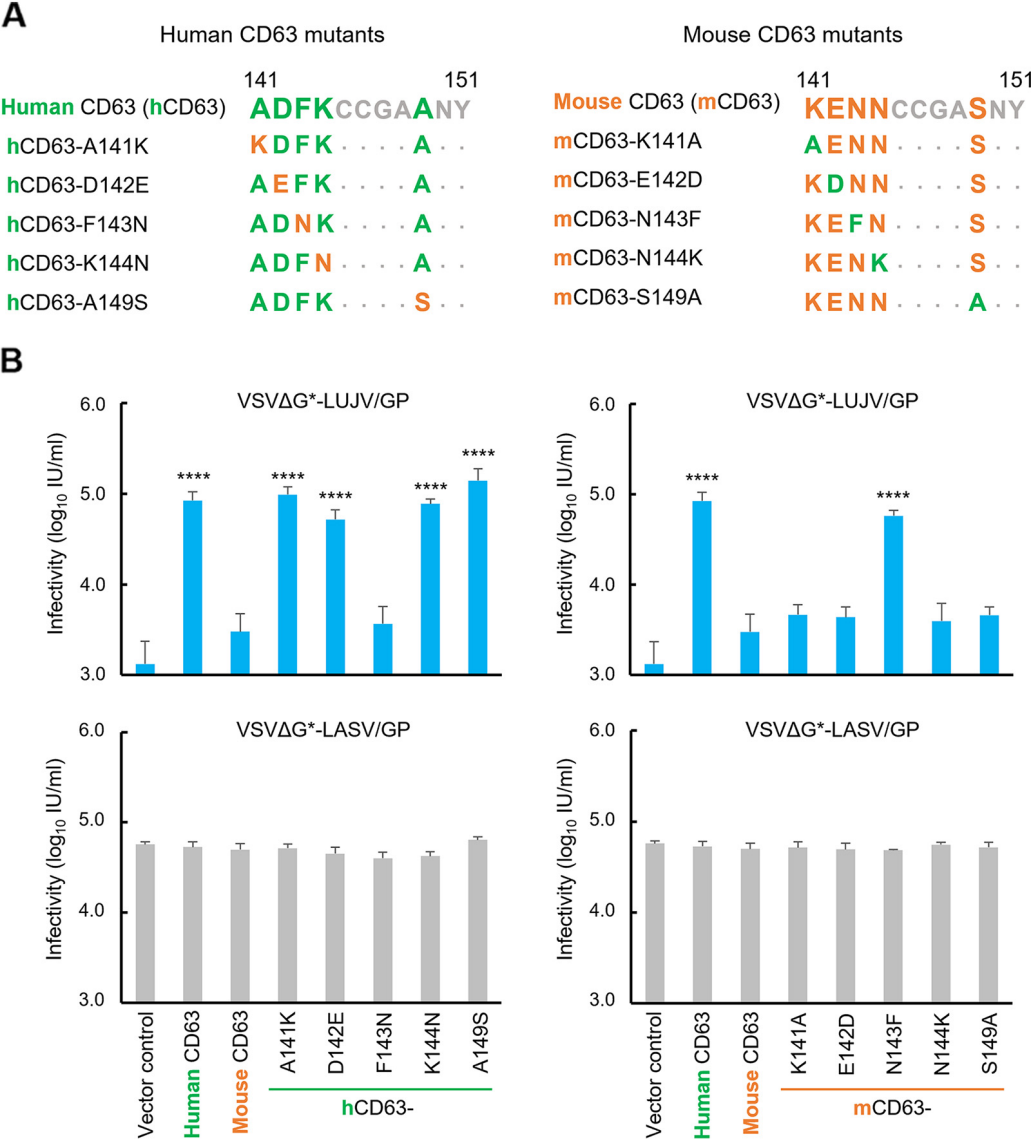
Importance of the amino acid residue at position 143 of human CD63 for VSVΔG*-LUJV/GP entry. (A) Amino acid sequences at positions 141 to 151 of the wild-type human, wild-type mouse, and mutant CD63s produced in this study. Amino acid residues shown in green and orange indicate those from human and mouse CD63s, respectively. (B) Infectious units (IU) of VSVΔG*-LUJV/GP or VSVΔG*-LASV/GP in BHK cells expressing wild-type human, wild-type mouse, and mutant CD63s. Each experiment was conducted three times, and averages and standard deviations are shown. Significant differences compared to the cells transduced with the vector control are shown (*, *P < *0.05; **, *P < *0.01; ***, *P < *0.001; ****, *P < *0.0001).

In the region from amino acids 141 to 150, there are 5 amino acid differences between human and mouse CD63s (Fig. 5A). To determine which amino acid residue(s) contributed to LUJV cellular entry, we generated CD63 mutants containing point mutations at each amino acid position. In these CD63 mutants, the different amino acid residues were swapped between human and mouse CD63s (Fig. 5A). Next, BHK cells stably expressing these CD63 mutants were produced, and their susceptibilities to VSVΔG*-LUJV/GP and VSVΔG*-LASV/GP were compared (Fig. 5B). The protein expression and intracellular localization were confirmed as described above (Fig. S5). As with human and mouse wild-type CD63s, the expression of all these CD63 mutants in BHK cells did not significantly affect the efficiency of VSVΔG*-LASV/GP entry (Fig. 5B, bottom). In contrast, swapping of the phenylalanine and asparagine residues at position 143 between human and mouse CD63s (F143N and N143F) completely exchanged the susceptibilities of the exogenous CD63-expressing cells to VSVΔG*-LUJV/GP (Fig. 5B, top). Similar results were obtained when NIH 3T3 cells were used for the expression of the CD63 molecules (Fig. S6). These results indicated that the amino acid at position 143 was responsible for the differential susceptibilities between the human and mouse cell lines (i.e., HEK293T and NIH 3T3) to VSVΔG*-LUJV/GP.

10.1128/mBio.03060-21.5 FIG S5 Expression of exogenous human and mouse CD63s and their mutants with single amino acid substitutions in BHK cells (related to Fig. 5). (A) The intracellular localization of CD63 in BHK cells was analyzed by confocal microscopy as described in Materials and Methods. Bars, 20 μm. (B) Each cell lysate was separated by SDS-PAGE followed by Western blotting as described in Materials and Methods. Download FIG S5, PDF file, 0.4 MB. Copyright © 2022 Saito et al. 2022 Saito et al. https://creativecommons.org/licenses/by/4.0/ This content is distributed under the terms of the Creative Commons Attribution 4.0 International license.

10.1128/mBio.03060-21.6 FIG S6 Importance of the amino acid residue at position 143 of human CD63 for VSVΔG*-LUJV/GP entry into NIH 3T3 cells (related to Fig. 5). The infectious units (IU) of VSVΔG*-LUJV/GP and VSVΔG*-LASV/GP in NIH 3T3 cells expressing wild-type human, mouse, or mutant CD63s are shown. Each experiment was conducted three times, and averages and standard deviations are shown. Significant differences compared to the cells transduced with the vector control (Vector control) are shown (*, *P* < 0.05; **, *P* < 0.01; ***, *P* < 0.001; ****, *P* < 0.0001). Download FIG S6, PDF file, 0.2 MB. Copyright © 2022 Saito et al. 2022 Saito et al. https://creativecommons.org/licenses/by/4.0/ This content is distributed under the terms of the Creative Commons Attribution 4.0 International license.

### Effects of the amino acid substitution at position 143 of CD63 on LUJV GP-mediated membrane fusion.

**FIG 6:**
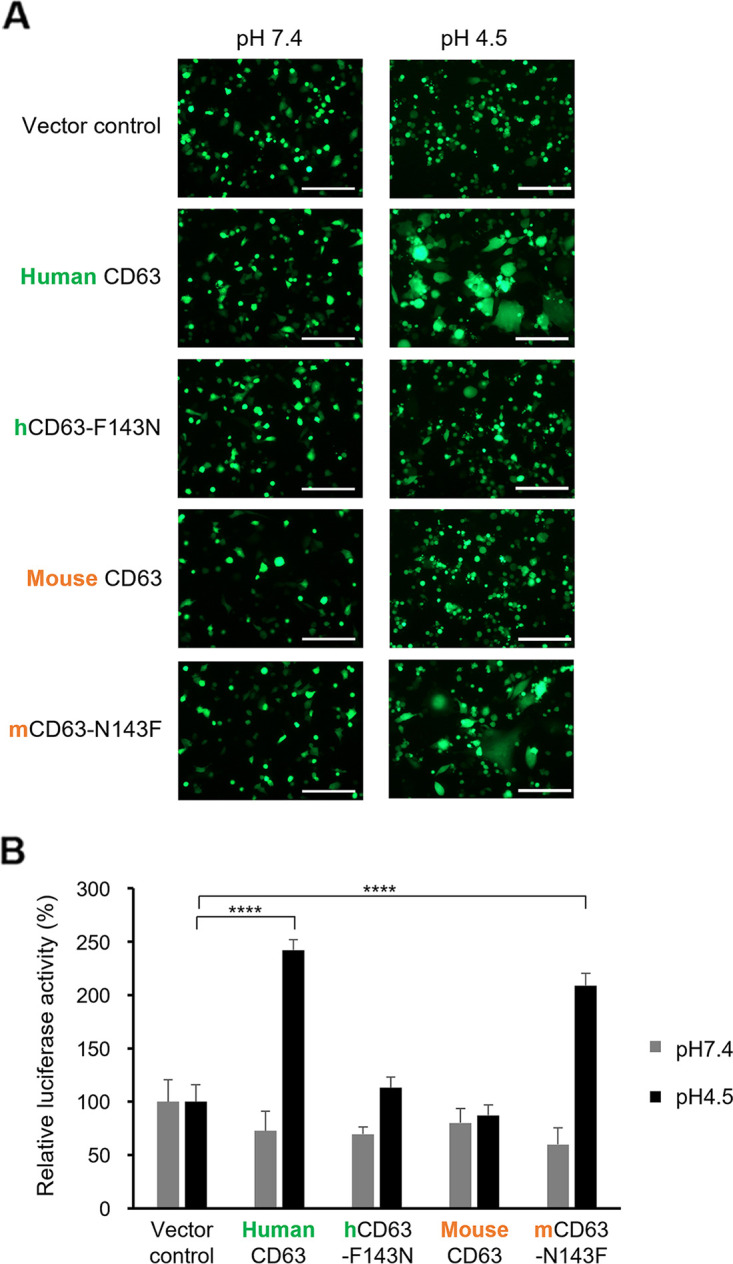
Importance of the amino acid residue at position 143 of human CD63 for LUJV GP-mediated cell fusion. (A) Syncytium formation assays for LUJV GP-expressing HEK293 cells after treatment with low- and neutral-pH buffers (pH 4.5 and 7.4, respectively). Syncytium formation was observed by fluorescence microscopy after a 24-h incubation. Bars, 200 μm. (B) Cell fusion activity quantitatively detected after exposure at low and neutral pH (4.5 and 7.4, respectively). Each experiment was conducted three times, and averages and standard deviations are shown. Relative luciferase activity was calculated under each pH condition, setting the value of the vector control to 100%. Significant differences compared to the vector control cells are shown (*, *P < *0.05; **, *P < *0.01; ***, *P < *0.001; ****, *P < *0.0001).

It has been thought that low-pH-activated LUJV GP is released from the attachment receptor NRP2 and then interacts with the endosomal fusion receptor CD63 (i.e., receptor switch), leading to membrane fusion. To directly investigate membrane fusion activity mediated by the interaction between LUJV GP and CD63, we performed a quantitative reporter fusion assay using HEK293 cells expressing LUJV GP and cell surface-localized exogenous CD63 (i.e., GY234AA mutant) (17) (Fig. 6 and Fig. S7). At neutral pH, appreciable syncytium formation was not detected in any of the CD63-expressing cells. In contrast, significantly increased syncytium formation associated with increased luciferase activity was observed at low pH in the cells expressing wild-type human CD63 or a mouse CD63 mutant having a single mutation (mCD63-N143F), whereas the cells expressing wild-type mouse CD63 or a human CD63 mutant with the converse mutation (hCD63-F143N) showed only limited fusion activity, like the vector control cells. These data indicated that the differential susceptibilities between the human and mouse cell lines was determined by the ability of CD63 to mediate membrane fusion and that the amino acid residue at position 143 played a major role in the interaction with LUJV GP.

10.1128/mBio.03060-21.7 FIG S7 Cell surface localization of exogenous CD63 in HEK293 cells shown in Fig. 6. The surface localizations of the exogenous human CD63 mutants (A) and mouse CD63 mutants (B) were confirmed by flow cytometric analyses. For these analyses, mouse anti-human CD63 (A) and rat anti-mouse CD63 (B) monoclonal antibodies were used as primary antibodies. Primary antibody binding was detected with goat Alexa Fluor 488-conjugated anti-mouse IgG and goat Alexa Fluor 488-conjugated anti-rat IgG antibodies. After washing, the cells were analyzed by employing a FACS Canto flow cytometer (BD Biosciences) and FlowJo software (TreeStar). Download FIG S7, PDF file, 0.5 MB. Copyright © 2022 Saito et al. 2022 Saito et al. https://creativecommons.org/licenses/by/4.0/ This content is distributed under the terms of the Creative Commons Attribution 4.0 International license.

## DISCUSSION

LUJV caused an outbreak in Zambia and South Africa in 2008 (1). The nosocomial transmission in this outbreak indicated that LUJV caused human-to-human transmission. Since then, patients with LUJV infection have never been reported, and LUJV has never been detected in any animal species (5, 6). Although LUJV is classified into the OW arenavirus group because of its discovery in Africa, it is phylogenetically distinct from the other OW arenaviruses, including LASV (1). In accordance with the structural difference of the GP1 molecule between LUJV and LASV, LUJV utilizes a unique cellular attachment factor, NRP2 (11). With pH reduction in the endosome, LASV and LUJV GPs switch their binding partners from attachment receptors to the intracellular fusion receptors lysosome-associated membrane protein 1 (LAMP1) and CD63, respectively (11, 18). It has been shown that LAMP1 is the key factor for LASV host restriction and that the chicken LAMP1 ortholog lacking the specific N-glycosylation site does not mediate LASV infection (18, 19). Thus, the difference in receptor usage has been suggested to affect the host ranges and natural host preferences of mammarenaviruses (20). In the present study, we found that VSVΔG*-LASV/GP infected the mouse and hamster cell lines more efficiently than VSVΔG*-LUJV/GP. It may be important to note that human and mouse LAMP1 share an N-glycosylation site that is critical for LASV infection (18, 19), and this could explain the relatively high susceptibility of the rodent cell lines to VSVΔG*-LASV/GP. On the other hand, we found that there were considerable amino acid differences between human and mouse CD63s and that the substantially low susceptibility of the mouse cell line to VSVΔG*-LUJV/GP was due to the reduced ability of mouse CD63 to mediate membrane fusion. Interestingly, the importance of the endosomal fusion receptor in differential host tropisms of filoviruses has also been reported (21–23). These observations imply a common concept explaining the molecular mechanism underlying viral host ranges controlled by their endosomal fusion receptors.

Our data suggest that LUJV GP interacts with the LEL region of CD63 and that the amino acid residues at positions 141 to 150 of human CD63, especially the phenylalanine residue at position 143, are essential for GP-mediated membrane fusion and, thus, LUJV entry into cells. We attempted to analyze the direct interaction between LUJV GP and CD63s by an immunoprecipitation assay but could not detect direct binding of these molecules (data not shown), consistent with a previous study (11). We assume that the binding affinity between LUJV GP and CD63 might be weak and that the presence of detergents during immunoprecipitation assays interfered with the binding of these molecules. However, our fusion assay strongly suggests that the interaction between LUJV GP and CD63 is crucial for mediating membrane fusion.

It should be noted that human CD63 having the amino acid region from positions 102 to 121 of mouse CD63 (chCD63-mLEL102–121) showed a reduced ability to mediate VSVΔG*-LUJV/GP entry, although its impact was less than that of the region from positions 141 to 150 (Fig. 4B). In contrast, mouse CD63 having the amino acid region from positions 102 to 121 of human CD63 (cmCD63-hLEL102–121) did not increase the susceptibility of the transduced BHK cells to VSVΔG*-LUJV/GP. This partial negative effect on human CD63 might be due to the difference in N-glycosylation motifs between human and mouse CD63. Mouse CD63, but not human CD63, contains an additional N-glycosylation motif in the region from positions 102 to 121 (positions 116 to 118, N-K-S) (Fig. 3). It is conceivable that the contact of LUJV GP with the phenylalanine at position 143 on chCD63-mLEL102–121 might have been interfered with this additional glycan derived from the mouse sequence from positions 102 to 121.

For the development of antivirals that inhibit viral entry into cells, it is important to clarify the structural basis and molecular mechanism of the interactions between viral surface proteins and their receptors. For example, the interaction between the E2 glycoprotein of hepatitis C virus (HCV) and CD81, another tetraspanin, has been well investigated (24, 25). The crystal structure of the complex of the HCV E2 glycoprotein and CD81 was resolved, and the receptor binding region of the E2 glycoprotein has been identified (26). The receptor binding regions of the E glycoprotein are targeted by multiple neutralizing antibodies (27). As antiviral drug candidates that inhibit the entry step of HCV, small chemical compounds mimicking the CD81 LEL partial structure were reported to block the binding of the E glycoprotein to CD81 (28, 29). As is the case with the interaction between CD81 and the HCV E2 glycoprotein, the molecular interface between the CD63 LEL region and LUJV GP may also be a target for neutralizing antibodies and antiviral drugs that block the entry of the virus into host cells.

**FIG 7:**
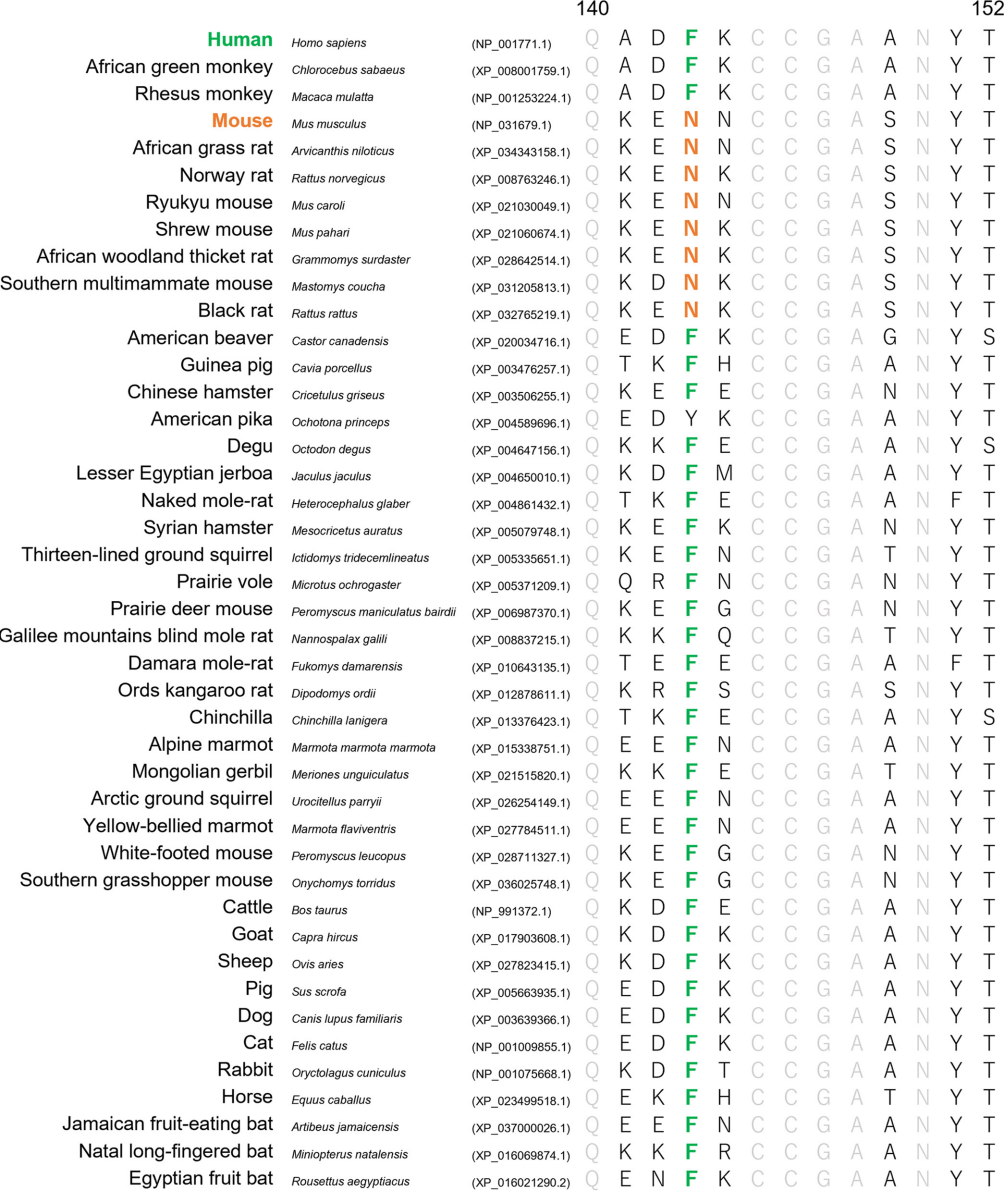
Comparison of amino acid sequences at positions 140 to 152 of CD63 orthologs from mammalian species. Amino acid sequences of CD63 orthologs were extracted from the NCBI database (https://www.ncbi.nlm.nih.gov/gene/967/ortholog/?scope=7776&term=CD63), and multiple-sequence alignment was performed by using the ClustalW program (human CD63 numbering). GenBank accession numbers are shown in parentheses. The phenylalanine at position 143 identical to that of human CD63 and the asparagine at the same position identical to that of mouse CD63 are indicated in green and orange, respectively. Amino acid residues shared among all the examined animal species are shown in gray. All of the sequences collected from the NCBI database are shown in Table S1 in the supplemental material.

Since LUJV infection was first reported in 2008, LUJV has not been found anywhere, and information on its host range and natural hosts has also been quite limited. Interestingly, the phenylalanine at position 143 (human CD63 numbering) of CD63 orthologs is conserved in many mammalian species (Fig. 7; see also Table S1 in the supplemental material). In general, natural hosts of mammarenaviruses are restricted by geographic and molecular factors, but our data imply that LUJV potentially has a larger host range than the other arenaviruses. On the other hand, some small rodent species such as Mus musculus (mouse), Arvicanthis niloticus (African grass rat), Grammomys surdaster (African woodland thicket rat), and Mastomys coucha (Southern multimammate mouse), which are the natural hosts of lymphocytic choriomeningitis virus, Ippy virus, Solwezi virus, and Lassa virus, respectively (30–33), have different amino acid residues at this position. It has been shown that laboratory mice are resistant to LUJV infection, whereas guinea pigs are highly susceptible to the virus, and the infection causes fatal disease (14). Guinea pig CD63 contains the human-type amino acid residue (phenylalanine) at position 143. Cynomolgus macaques, which have phenylalanine at position 143, are also susceptible to LUJV, although they do not develop lethal disease (13). These findings may explain the susceptibility of these animal species to LUJV. However, given that viruses that cause severe diseases in humans and some animals are generally thought to be nonpathogenic in their natural hosts, the possibility of differential receptor usage in natural hosts is not ruled out.

10.1128/mBio.03060-21.8 TABLE S1 Comparison of amino acid sequences among animals (related to Fig. 7). Download Table S1, PDF file, 0.3 MB. Copyright © 2022 Saito et al. 2022 Saito et al. https://creativecommons.org/licenses/by/4.0/ This content is distributed under the terms of the Creative Commons Attribution 4.0 International license.

On the other hand, our data suggest that hamster CD63 is not fully capable of mediating LUJV entry, although it has a phenylalanine at this amino acid position. To investigate the contribution of the amino acid region from positions 141 to 150 of hamster CD63, we produced transduced cells expressing chimeric human CD63s having the amino acid region from positions 141 to 150 from the hamster and investigated their susceptibilities to VSVΔG*-LUJV/GP (data not shown). The cells expressing the chimeric human CD63 having the amino acid region from positions 141 to 150 of hamster CD63 were found to have retained susceptibility to VSVΔG*-LUJV/GP. This suggests that another region(s) of CD63 might also be involved in the reduced susceptibility of BHK cells to VSVΔG*-LUJV/GP.

In this report, we focused on the distinct susceptibilities of human- and mouse-derived cell lines to VSVΔG*-LUJV/GP and identified a molecular factor that is important for the interaction between LUJV GP and CD63 as well as for the host tropism of LUJV. Information on the host range and natural hosts of LUJV is a prerequisite for the establishment of epidemiological control strategies, and elucidation of the molecular mechanisms of LUJV entry into cells is critical for the development of effective anti-LUJV drugs. Our data provide important information on the possible host range of LUJV and on a potential drug target that inhibits LUJV entry into cells.

## MATERIALS AND METHODS

### Cell lines.

African green monkey kidney Vero E6, human hepatoma Huh7, human embryonic kidney HEK293T and HEK293, murine embryo fibroblast NIH 3T3, and baby hamster kidney fibroblast BHK cells were grown in Dulbecco’s modified Eagle’s medium (DMEM) (Sigma-Aldrich) supplemented with 10% fetal calf serum (FCS) (Cell Culture Bioscience), 100 U/mL penicillin, and 0.1 mg/mL streptomycin (Gibco). Plat-GP cells (Cell Biolabs) were grown in DMEM supplemented with 10% FCS. All the cell lines were grown at 37°C in a 5% CO_2_ incubator.

### Viruses.

Vesicular stomatitis virus (VSV) (Indiana) containing the green fluorescent protein (GFP) gene instead of the VSV glycoprotein (G) gene (VSVΔG*-G) and pseudotyped VSVs bearing GPs of LASV (strain Josiah) or LUJV (strain IGR140) were generated as described previously (15, 34). Briefly, HEK293T cells were transfected with the expression plasmid pCAGGS carrying the LUJV GP gene, and 24 h later, the cells were infected with VSVΔG*-G at a multiplicity of infection of 1.0. After a 16-h incubation, the supernatants were collected and centrifuged to remove cell debris. Infectious units (IU) of the pseudotyped VSVs in each cell line were determined as described previously (35, 36). Briefly, cells were seeded into 96-well plates 1 day before virus inoculation, and 24 h later, GFP-positive cells were counted using an In Cell analyzer 2000 system (GE Healthcare). To reduce the background infectivity of the residual parent VSVΔG*-G, each pseudotyped virus stock was treated with a neutralizing monoclonal antibody specific for the VSV G protein (VSV-G[N]1-9) before use (37).

### Construction of plasmids and generation of cells expressing CD63 and/or NRP2.

Total RNA was extracted from HEK293T, NIH 3T3, and BHK cells using Isogen (Nippongene), and mRNAs were reverse transcribed with Superscript IV (Invitrogen). To amplify the CD63 and NRP2 genes, PCR was performed with KOD-One (Toyobo) using primer sets designed based on the sequences of human CD63 (GenBank accession number NM_001257389.1), mouse CD63 (GenBank accession number NM_001042580.1), hamster CD63 (GenBank accession number XM_013120354.2), and human NRP2 (GenBank accession number NM_201266.1). The cDNA encoding human CD63 fused with an N-terminal 3×FLAG tag was cloned into the pMXs-neo vector (Cell Biolabs) using an In-Fusion cloning kit (BD Clontech), and the cDNA encoding human NRP2 fused with a C-terminal hemagglutinin (HA) tag was also cloned into the pMXs-puro vector (Cell Biolabs). In the same way, the cDNAs encoding mouse CD63 and hamster CD63 were also cloned into the pMXs-neo vector. The cDNAs encoding chimeric CD63 and point mutant CD63 were amplified using KOD-One (Toyobo) and the primers containing the desired mutations and cloned into the pMXs-neo vectors using an In-Fusion HD cloning kit (Clontech). To generate the retroviruses carrying the CD63 or NRP2 genes, Plat-GP cells were transfected with the pMXs-neo or pMXs-puro vectors carrying cDNAs of these genes together with the expression plasmid pCAGGS carrying the VSV G gene using the TransIT-LT1 transfection reagent (Mirus) according to the manufacturer’s instructions. Two days after transfection, the culture supernatants were collected, filtered with 0.45-μm filters, and inoculated into BHK cells and NIH 3T3 cells. BHK cells and NIH 3T3 cells stably expressing CD63 or NRP2 were selected with DMEM containing 10% FCS and 1,000 μg/mL G418 (GE Healthcare) or 10 μg/mL puromycin (InvivoGen). BHK cells and NIH 3T3 cells stably expressing both human CD63 and NRP2 were generated by infecting BHK cells and NIH 3T3 cells with both retroviruses carrying human CD63 and NRP2 and selected with both 1,000 μg/mL G418 and 10 μg/mL puromycin. The expression of exogenous CD63 and NRP2 was confirmed by Western blotting. For vector control cells, BHK cells and NIH 3T3 cells were transduced with the retrovirus produced with the pMXs-neo vector alone.

### Sodium dodecyl sulfate-polyacrylamide gel electrophoresis and Western blotting.

Cells (6.0 × 10^5^ cells for each cell line) were lysed with 100 μL radioimmunoprecipitation assay (RIPA) buffer (50 mM Tris-HCl [pH 7.4], 150 mM NaCl, 1% NP-40, 0.5% sodium deoxycholate, and 0.1% sodium dodecyl sulfate [SDS]) containing a protease inhibitor mixture (Thermo Scientific) and incubated for 30 min on ice. After centrifugation (10,000 × *g* at 4°C) for 10 min, the supernatants were mixed with SDS-polyacrylamide gel electrophoresis (PAGE) sample buffer (Bio-Rad) containing 10% 2-mercaptoethanol and incubated for 5 min at 98°C. The lysed proteins were separated in 6% (for NRP2) or 10% (for CD63 and β-actin) SDS-PAGE gels and transferred to a polyvinylidene difluoride (PVDF) membrane (Merck). Phosphate-buffered saline (PBS) containing 5% skim milk (BD) and PBS containing 0.05% Tween 20 (PBST) were used as blocking and wash buffers, respectively. The PVDF membrane was incubated with a mouse anti-FLAG M2 monoclonal antibody (Sigma-Aldrich), a goat anti-NRP2 polyclonal antibody (R&D Systems), or a mouse anti-β-actin monoclonal antibody (Abcam) for 60 min; washed with PBST; and then incubated with a goat anti-mouse IgG polyclonal antibody conjugated with horseradish peroxidase (HRP) (Jackson ImmunoResearch) or a donkey anti-goat IgG polyclonal antibody conjugated with HRP (Jackson ImmunoResearch) for 60 min. After washing with PBST, the bound antibodies were visualized with Immobilon Western (Millipore) and an Amersham Imager 600 system (GE Healthcare).

### Immunofluorescence assay.

To investigate the expression and localization of exogenous CD63, BHK cells expressing exogenous 3×FLAG-tagged CD63 were seeded into eight-well chamber slides (Merck) precoated with poly-l-lysine (Cultrex). Twenty-four hours after cell seeding, the cells were washed with ice-cold PBS, fixed, and permeabilized with ice-cold methanol for 15 min. After washing with PBS, the cells were incubated with PBST containing 3% bovine serum albumin for blocking, followed by incubation with the mouse anti-FLAG M2 monoclonal antibody (Sigma-Aldrich) and a rabbit anti-lysosome-associated membrane protein 1 (LAMP1) polyclonal antibody (Sigma-Aldrich) as a late endosome marker for 1 h at room temperature. The cells were washed with PBST and then incubated with a goat Alexa Fluor 488-conjugated anti-mouse IgG antibody (Thermo Fisher), a goat Alexa Fluor 594-conjugated anti-rabbit IgG antibody (Thermo Fisher), and 5 μg/mL Hoechst 33342 (Thermo Fisher) for 1 h in the dark at room temperature. Images were acquired with a 100× oil lens objective on a Zeiss LSM700 inverted microscope using ZEN 2009 software (Carl Zeiss).

### Fusion assay.

To increase the cell surface localization of CD63 exogenously introduced into HEK293 cells, retroviruses expressing CD63 with a mutated C-terminal lysosomal targeting motif (GY234AA) (17) were generated, and HEK293 cells were transduced with the viruses. The surface localization of the exogenous CD63 was confirmed by flow cytometric analysis (see Fig. S7 in the supplemental material). For this analysis, a mouse anti-human CD63 monoclonal antibody (Fujifilm Wako Pure Chemical Corporation) and a rat anti-mouse CD63 monoclonal antibody (MBL) as primary antibodies and a goat Alexa Fluor 488-conjugated anti-mouse IgG antibody (Thermo Fisher) and a goat Alexa Fluor 488-conjugated anti-rat IgG antibody (Thermo Fisher) as secondary antibodies were used. As the target cells, 1.5 × 10^5^ HEK293 cells were cultured in poly-l-lysine-coated 96-well plates, and 6 h later, the cells were transfected with pCAGGS carrying the LUJV GP gene and pTOPO-T7-IRES-nanoluciferase using TransIT-LT1 transfection reagent (Mirus) according to the manufacturer’s instructions. The expression of surface LUJV GP was confirmed by flow cytometry using an in-house mouse anti-LUJV GP monoclonal antibody. As the effector cells, 3.0 × 10^5^ HEK293 cells stably expressing exogenous CD63 were seeded into 6-well plates 6 h prior to transfection. As vector control cells, HEK293 cells transduced with the retrovirus produced with the pMXs-neo vector were used. The effector HEK293 cells were transfected with pCAGGS plasmids carrying the GFP and pCAGGS-T7 polymerase genes using TransIT-LT1. Twenty-four hours after transfection, the effector cells were harvested with trypsin-EDTA and resuspended in DMEM supplemented with 10% FCS, and 3.0 × 10^4^ cells were then overlaid onto the target cells in each well of 96-well plates. After coculturing target and effector cells for 24 h, the cells were washed once with PBS and then treated with citrate-phosphate buffer (pH 4.5) or PBS (pH 7.4) for 10 min at 37°C, followed by washing with PBS and replacement of the medium with DMEM containing 10% FCS. After a 24-h incubation, syncytium formation was examined by fluorescence microscopy, and fusion activity was quantified by measuring the relative luciferase activity using the Nano-Glo luciferase assay system (Promega) according to the manufacturer’s instructions.

### Multiple alignment of amino acid sequences of CD63 orthologs.

All sequences identified in this study were obtained from the NCBI database (https://www.ncbi.nlm.nih.gov/gene/967/ortholog/?scope=7776&term=CD63). The multiple-sequence alignment of CD63 orthologs was performed using ClustalW on the Kyoto University Bioinformatics Center website (https://www.genome.jp/tools-bin/clustalw). The alignments of amino acid sequences at positions 140 to 152 of CD63 orthologs from mammalian species are shown in Table S1.

### Quantification and statistical analysis.

All data were analyzed using GraphPad Prism version 6.0 software. For comparison of viral infectivity among CD63-transduced cell lines, we performed one-way repeated-measures analysis of variance (ANOVA), followed by Dunnett’s test. *P* values of less than 0.05 were considered statistically significant.

## References

[1] Briese T, Paweska JT, McMullan LK, Hutchison SK, Street C, Palacios G, Khristova ML, Weyer J, Swanepoel R, Egholm M, Nichol ST, Lipkin WI. 2009. Genetic detection and characterization of Lujo virus, a new hemorrhagic fever-associated arenavirus from southern Africa. PLoS Pathog 5:e1000455. doi:10.1371/journal.ppat.1000455. 19478873 PMC2680969

[2] Moraz ML, Kunz S. 2011. Pathogenesis of arenavirus hemorrhagic fevers. Expert Rev Anti Infect Ther 9:49–59. doi:10.1586/eri.10.142. 21171877

[3] Sewlall NH, Richards G, Duse A, Swanepoel R, Paweska J, Blumberg L, Dinh TH, Bausch D. 2014. Clinical features and patient management of Lujo hemorrhagic fever. PLoS Negl Trop Dis 8:e3233. doi:10.1371/journal.pntd.0003233. 25393244 PMC4230886

[4] Downs WG, Anderson CR, Spence L, Aitken TH, Greenhall AH. 1963. Tacaribe virus, a new agent isolated from Artibeus bats and mosquitoes in Trinidad, West Indies. Am J Trop Med Hyg 12:640–646. doi:10.4269/ajtmh.1963.12.640. 22324073

[5] Ishii A, Thomas Y, Moonga L, Nakamura I, Ohnuma A, Hang’ombe B, Takada A, Mweene A, Sawa H. 2011. Novel arenavirus, Zambia. Emerg Infect Dis 17:1921–1924. doi:10.3201/eid1710.10452. 22000372 PMC3310648

[6] Simulundu E, Mweene AS, Changula K, Monze M, Chizema E, Mwaba P, Takada A, Ippolito G, Kasolo F, Zumla A, Bates M. 2016. Lujo viral hemorrhagic fever: considering diagnostic capacity and preparedness in the wake of recent Ebola and Zika virus outbreaks. Rev Med Virol 26:446–454. doi:10.1002/rmv.1903. 27593704 PMC7169100

[7] Hallam SJ, Koma T, Maruyama J, Paessler S. 2018. Review of mammarenavirus biology and replication. Front Microbiol 9:1751. doi:10.3389/fmicb.2018.01751. 30123198 PMC6085440

[8] Oppliger J, da Palma JR, Burri DJ, Bergeron E, Khatib A-M, Spiropoulou CF, Pasquato A, Kunz S. 2016. A molecular sensor to characterize arenavirus envelope glycoprotein cleavage by subtilisin kexin isozyme 1/site 1 protease. J Virol 90:705–714. doi:10.1128/JVI.01751-15. 26512085 PMC4702697

[9] Urata S, Weyer J, Storm N, Miyazaki Y, van Vuren PJ, Paweska JT, Yasuda J. 2016. Analysis of assembly and budding of Lujo virus. J Virol 90:3257–3261. doi:10.1128/JVI.03198-15. PMC4810620 26719243

[10] Rojek JM, Kunz S. 2008. Cell entry by human pathogenic arenaviruses. Cell Microbiol 10:828–835. doi:10.1111/j.1462-5822.2007.01113.x. 18182084

[11] Raaben M, Jae LT, Herbert AS, Kuehne AI, Stubbs SH, Chou Y-Y, Blomen VA, Kirchhausen T, Dye JM, Brummelkamp TR, Whelan SP. 2017. NRP2 and CD63 are host factors for Lujo virus cell entry. Cell Host Microbe 22:688–696.e5. doi:10.1016/j.chom.2017.10.002. 29120745 PMC5821226

[12] Cohen-Dvashi H, Kilimnik I, Diskin R. 2018. Structural basis for receptor recognition by Lujo virus. Nat Microbiol 3:1153–1160. doi:10.1038/s41564-018-0224-5. 30150732

[13] Rasmussen AL, Tchitchek N, Safronetz D, Carter VS, Williams CM, Haddock E, Korth MJ, Feldmann H, Katze MG. 2015. Delayed inflammatory and cell death responses are associated with reduced pathogenicity in Lujo virus-infected cynomolgus macaques. J Virol 89:2543–2552. doi:10.1128/JVI.02246-14. 25520505 PMC4325716

[14] Bird BH, Dodd KA, Erickson BR, Albariño CG, Chakrabarti AK, McMullan LK, Bergeron E, Ströeher U, Cannon D, Martin B, Coleman-McCray JAD, Nichol ST, Spiropoulou CF. 2012. Severe hemorrhagic fever in strain 13/N guinea pigs infected with Lujo virus. PLoS Negl Trop Dis 6:e1801. doi:10.1371/journal.pntd.0001801. 22953019 PMC3429401

[15] Tani H, Iha K, Shimojima M, Fukushi S, Taniguchi S, Yoshikawa T, Kawaoka Y, Nakasone N, Ninomiya H, Saijo M, Morikawa S. 2014. Analysis of Lujo virus cell entry using pseudotype vesicular stomatitis virus. J Virol 88:7317–7330. doi:10.1128/JVI.00512-14. 24741091 PMC4054455

[16] Pols MS, Klumperman J. 2009. Trafficking and function of the tetraspanin CD63. Exp Cell Res 315:1584–1592. doi:10.1016/j.yexcr.2008.09.020. 18930046

[17] Latysheva N, Muratov G, Rajesh S, Padgett M, Hotchin NA, Overduin M, Berditchevski F. 2006. Syntenin-1 is a new component of tetraspanin-enriched microdomains: mechanisms and consequences of the interaction of syntenin-1 with CD63. Mol Cell Biol 26:7707–7718. doi:10.1128/MCB.00849-06. 16908530 PMC1636879

[18] Jae LT, Brummelkamp TR. 2015. Emerging intracellular receptors for hemorrhagic fever viruses. Trends Microbiol 23:392–400. doi:10.1016/j.tim.2015.04.006. 26004032

[19] Jae LT, Raaben M, Herbert AS, Kuehne AI, Wirchnianski AS, Soh TK, Stubbs SH, Janssen H, Damme M, Saftig P, Whelan SP, Dye JM, Brummelkamp TR. 2014. Lassa virus entry requires a trigger-induced receptor switch. Science 344:1506–1510. doi:10.1126/science.1252480. 24970085 PMC4239993

[20] Charrel RN, Lemasson JJ, Garbutt M, Khelifa R, De Micco P, Feldmann H, De Lamballerie X. 2003. New insights into the evolutionary relationships between arenaviruses provided by comparative analysis of small and large segment sequences. Virology 317:191–196. doi:10.1016/j.virol.2003.08.016. 14698659

[21] Ng M, Ndungo E, Kaczmarek ME, Herbert AS, Binger T, Kuehne AI, Jangra RK, Hawkins JA, Gifford RJ, Biswas R, Demogines A, James RM, Yu M, Brummelkamp TR, Drosten C, Wang L-F, Kuhn JH, Müller MA, Dye JM, Sawyer SL, Chandran K. 2015. Filovirus receptor NPC1 contributes to species-specific patterns of ebolavirus susceptibility in bats. Elife 4:e11785. doi:10.7554/eLife.11785. 26698106 PMC4709267

[22] Takadate Y, Kondoh T, Igarashi M, Maruyama J, Manzoor R, Ogawa H, Kajihara M, Furuyama W, Sato M, Miyamoto H, Yoshida R, Hill TE, Freiberg AN, Feldmann H, Marzi A, Takada A. 2020. Niemann-Pick C1 heterogeneity of bat cells controls filovirus tropism. Cell Rep 30:308–319.e5. doi:10.1016/j.celrep.2019.12.042. 31940478 PMC11075117

[23] Takadate Y, Manzoor R, Saito T, Kida Y, Maruyama J, Kondoh T, Miyamoto H, Ogawa H, Kajihara M, Igarashi M, Takada A. 2020. Receptor-mediated host cell preference of a bat-derived filovirus, lloviu virus. Microorganisms 8:1530. doi:10.3390/microorganisms8101530. PMC7601172 33027954

[24] Pileri P, Uematsu Y, Campagnoli S, Galli G, Falugi F, Petracca R, Weiner AJ, Houghton M, Rosa D, Grandi G, Abrignani S. 1998. Binding of hepatitis C virus to CD81. Science 282:938–941. doi:10.1126/science.282.5390.938. 9794763

[25] Zona L, Tawar RG, Zeisel MB, Xiao F, Schuster C, Lupberger J, Baumert TF. 2014. CD81-receptor associations—impact for hepatitis C virus entry and antiviral therapies. Viruses 6:875–892. doi:10.3390/v6020875. 24553110 PMC3939486

[26] Yang W, Zhang M, Chi X, Liu X, Qin B, Cui S. 2015. An intramolecular bond at cluster of differentiation 81 ectodomain is important for hepatitis C virus entry. FASEB J 29:4214–4226. doi:10.1096/fj.15-272880. 26116703

[27] Kinchen VJ, Zahid MN, Flyak AI, Soliman MG, Learn GH, Wang S, Davidson E, Doranz BJ, Ray SC, Cox AL, Crowe JE, Bjorkman PJ, Shaw GM, Bailey JR. 2018. Broadly neutralizing antibody mediated clearance of human hepatitis C virus infection. Cell Host Microbe 24:717–730.e5. doi:10.1016/j.chom.2018.10.012. 30439341 PMC6250073

[28] VanCompernolle SE, Wiznycia AV, Rush JR, Dhanasekaran M, Baures PW, Todd SC. 2003. Small molecule inhibition of hepatitis C virus E2 binding to CD81. Virology 314:371–380. doi:10.1016/S0042-6822(03)00406-9. 14517089

[29] Al Olaby RR, Cocquerel L, Zemla A, Saas L, Dubuisson J, Vielmetter J, Marcotrigiano J, Khan AG, Catalan FV, Perryman AL, Freundlich JS, Forli S, Levy S, Balhorn R, Azzazy HM. 2014. Identification of a novel drug lead that inhibits HCV infection and cell-to-cell transmission by targeting the HCV E2 glycoprotein. PLoS One 9:e111333. doi:10.1371/journal.pone.0111333. 25357246 PMC4214736

[30] Pontremoli C, Forni D, Sironi M. 2019. Arenavirus genomics: novel insights into viral diversity, origin, and evolution. Curr Opin Virol 34:18–28. doi:10.1016/j.coviro.2018.11.001. 30497052

[31] Swanepoel R, Leman PA, Shepherd AJ, Shepherd SP, Kiley MP, McCormick JB. 1985. Identification of Ippy as a Lassa-fever-related virus. Lancet i:639. doi:10.1016/S0140-6736(85)92175-0. 2857974

[32] Olayemi A, Fichet-Calvet E. 2020. Systematics, ecology, and host switching: attributes affecting emergence of the Lassa virus in rodents across western Africa. Viruses 12:312. doi:10.3390/v12030312. PMC7150792 32183319

[33] Těšíková J, Krásová J, Goüy de Bellocq J. 2021. Multiple mammarenaviruses circulating in Angolan rodents. Viruses 13:982. doi:10.3390/v13060982. 34070551 PMC8227972

[34] Takada A, Robison C, Goto H, Sanchez A, Murti KG, Whitt MA, Kawaoka Y. 1997. A system for functional analysis of Ebola virus glycoprotein. Proc Natl Acad Sci USA 94:14764–14769. doi:10.1073/pnas.94.26.14764. 9405687 PMC25111

[35] Furuyama W, Marzi A, Nanbo A, Haddock E, Maruyama J, Miyamoto H, Igarashi M, Yoshida R, Noyori O, Feldmann H, Takada A. 2016. Discovery of an antibody for pan-ebolavirus therapy. Sci Rep 6:20514. doi:10.1038/srep20514. 26861827 PMC4748290

[36] Maruyama J, Miyamoto H, Kajihara M, Ogawa H, Maeda K, Sakoda Y, Yoshida R, Takada A. 2014. Characterization of the envelope glycoprotein of a novel filovirus, lloviu virus. J Virol 88:99–109. doi:10.1128/JVI.02265-13. 24131711 PMC3911722

[37] Nakayama E, Tomabechi D, Matsuno K, Kishida N, Yoshida R, Feldmann H, Takada A. 2011. Antibody-dependent enhancement of Marburg virus infection. J Infect Dis 204:S978–S985. doi:10.1093/infdis/jir334. 21987779 PMC3189991

