# Supplementary material for: Molecular Mechanisms Underlying the Cellular Entry and Host Range Restriction of Lujo Virus
Source: mBio. 2022 Feb 15;13(1):e03060-21. doi: 10.1128/mbio.03060-21 (PMC8844913; doi:10.1128/mbio.03060-21)
Supplement: FIG S1 [file mbio.03060-21-sf001.pdf]

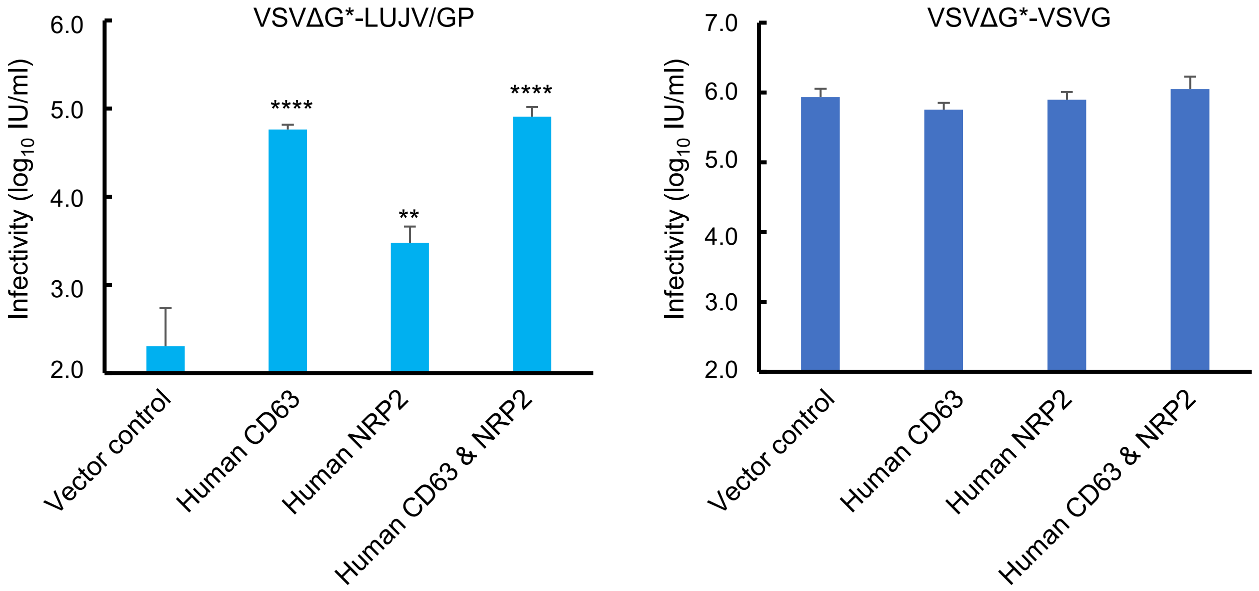

Figure S1. Figure S1. Importance of human CD63 expression for LUJV entry into mouse-derived cells (NIH3T3), Related to Figure 1.

NIH3T3 cells transduced with exogenous human CD63 and/or NRP2 genes were infected with VSVΔG*-LUJV/GP and VSVΔG*-G and infectious units (IU) were determined. Each experiment was conducted three times, and averages and standard deviations are shown. Significant differences compared to the cells transduced with the vector control (Vector control) are shown (**P* < 0.05, ***P* < 0.01, ****P* < 0.001, *****P*<0.0001).

Figure S1
