# Supplementary material for: Molecular Mechanisms Underlying the Cellular Entry and Host Range Restriction of Lujo Virus
Source: mBio. 2022 Feb 15;13(1):e03060-21. doi: 10.1128/mbio.03060-21 (PMC8844913; doi:10.1128/mbio.03060-21)
Supplement: FIG S2 [file mbio.03060-21-sf002.pdf]

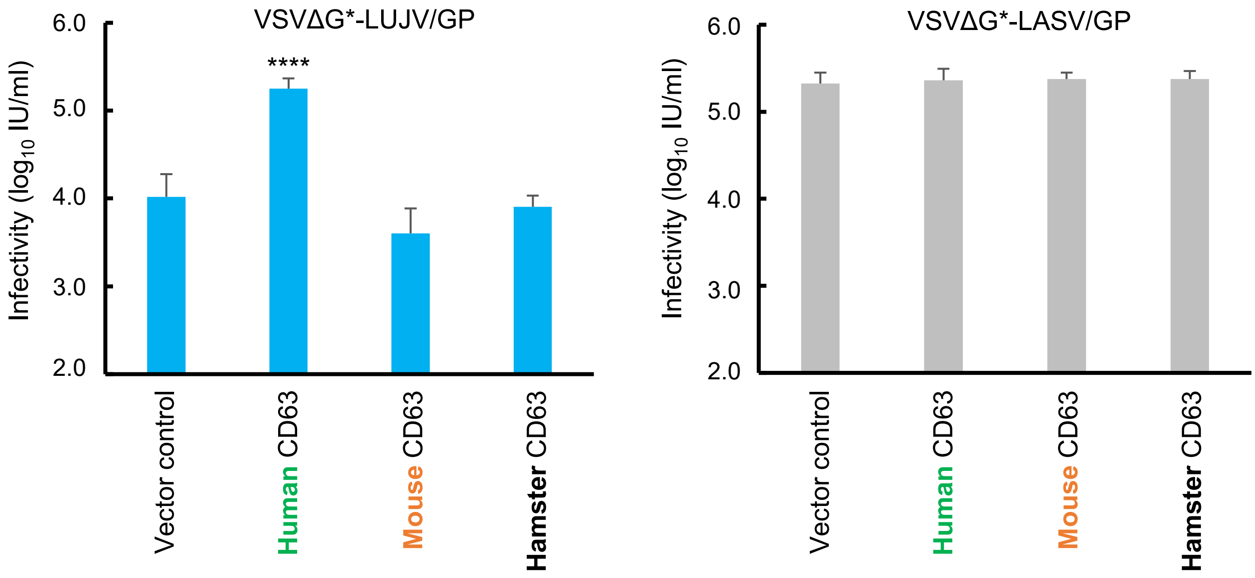

Figure S2. Effects of exogenous human, mouse, and hamster CD63 expression to infectivities of VSVΔG*-LUJV/GP and VSVΔG*-LASV/GP in NIH3T3 cells, Related to Figure 2.

Exogenous human, mouse, or hamster CD63-expressing NIH3T3 cells were infected with VSVΔG*-LUJV/GP or VSVΔG*-LASV/GP and infectious units (IU) were determined. Each experiment was conducted three times, and averages and standard deviations are shown. Significant differences compared to the cells transduced with the vector control (Vector control) are shown (*$P < 0.05$, **$P < 0.01$, ***$P < 0.001$, ****P<0.0001).

Figure S2
