# Supplementary material for: Molecular Mechanisms Underlying the Cellular Entry and Host Range Restriction of Lujo Virus
Source: mBio. 2022 Feb 15;13(1):e03060-21. doi: 10.1128/mbio.03060-21 (PMC8844913; doi:10.1128/mbio.03060-21)
Supplement: FIG S3 [file mbio.03060-21-sf003.pdf]

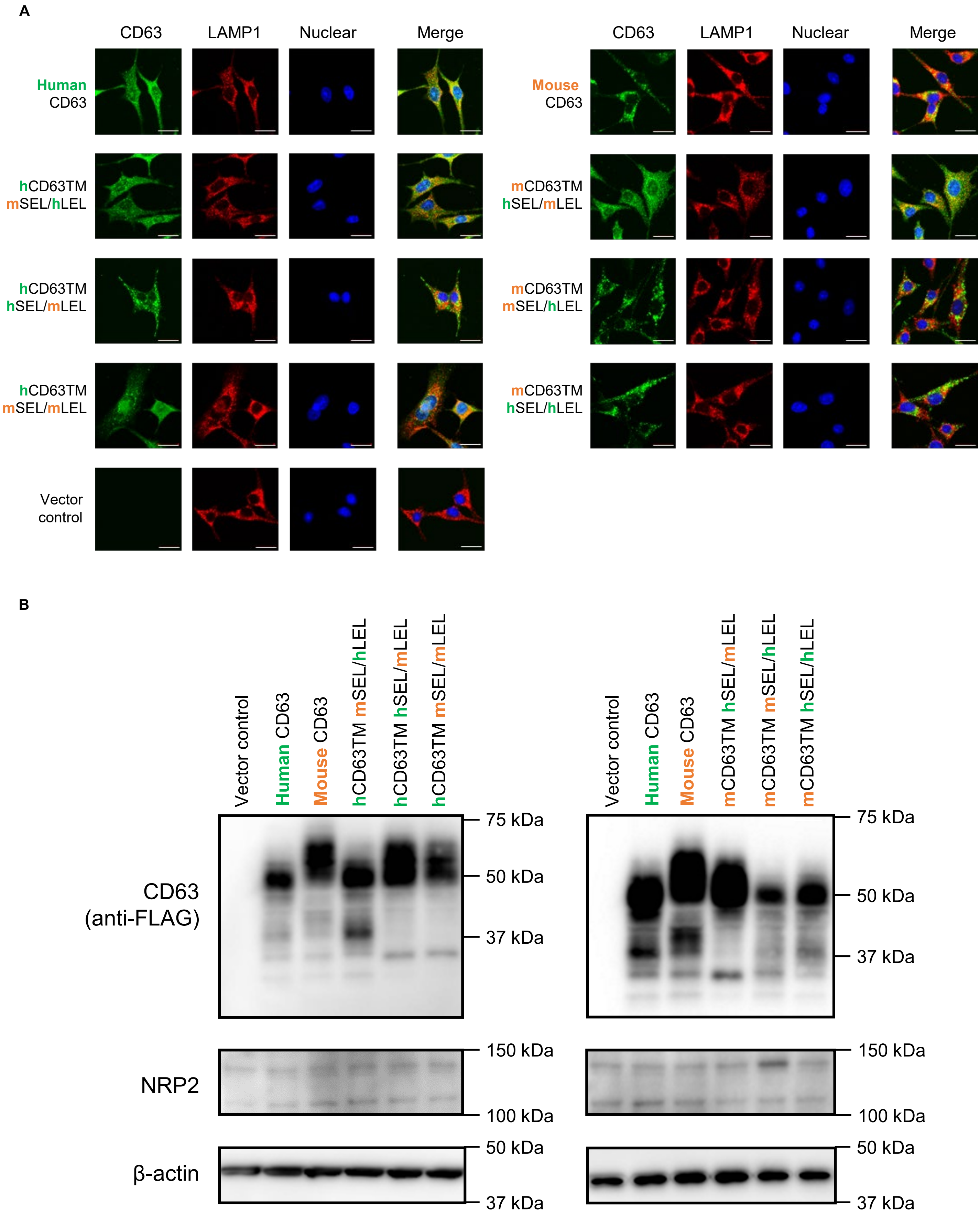
A
CD63
LAMP1
Nuclear
Merge
Human CD63
hCD63TM mSEL/hLEL
hCD63TM hSEL/mLEL
hCD63TM mSEL/mLEL
Vector control
Mouse CD63
mCD63TM hSEL/mLEL
mCD63TM mSEL/hLEL
mCD63TM hSEL/hLEL
B
Vector control
Human CD63
Mouse CD63
hCD63TM mSEL/hLEL
hCD63TM hSEL/mLEL
hCD63TM mSEL/mLEL
mCD63TM hSEL/mLEL
mCD63TM mSEL/hLEL
mCD63TM hSEL/hLEL
CD63 (anti-FLAG)
NRP2
β-actin
75 kDa
50 kDa
37 kDa
150 kDa
100 kDa

Figure S3

Figure S3. Expression of exogenous human and mouse CD63s and their chimeric mutants in BHK cells, Related to Figure 3.

(A) The intracellular localization of exogenous chimeric CD63 in BHK cells was analyzed by confocal microscopy as described in Materials and Methods. The scale bars represent 20 μm. (B) Each cell lysate was separated in SDS-PAGE followed by western blotting as described in Materials and Methods.
