# Supplementary material for: Molecular Mechanisms Underlying the Cellular Entry and Host Range Restriction of Lujo Virus
Source: mBio. 2022 Feb 15;13(1):e03060-21. doi: 10.1128/mbio.03060-21 (PMC8844913; doi:10.1128/mbio.03060-21)
Supplement: FIG S4 [file mbio.03060-21-sf004.pdf]

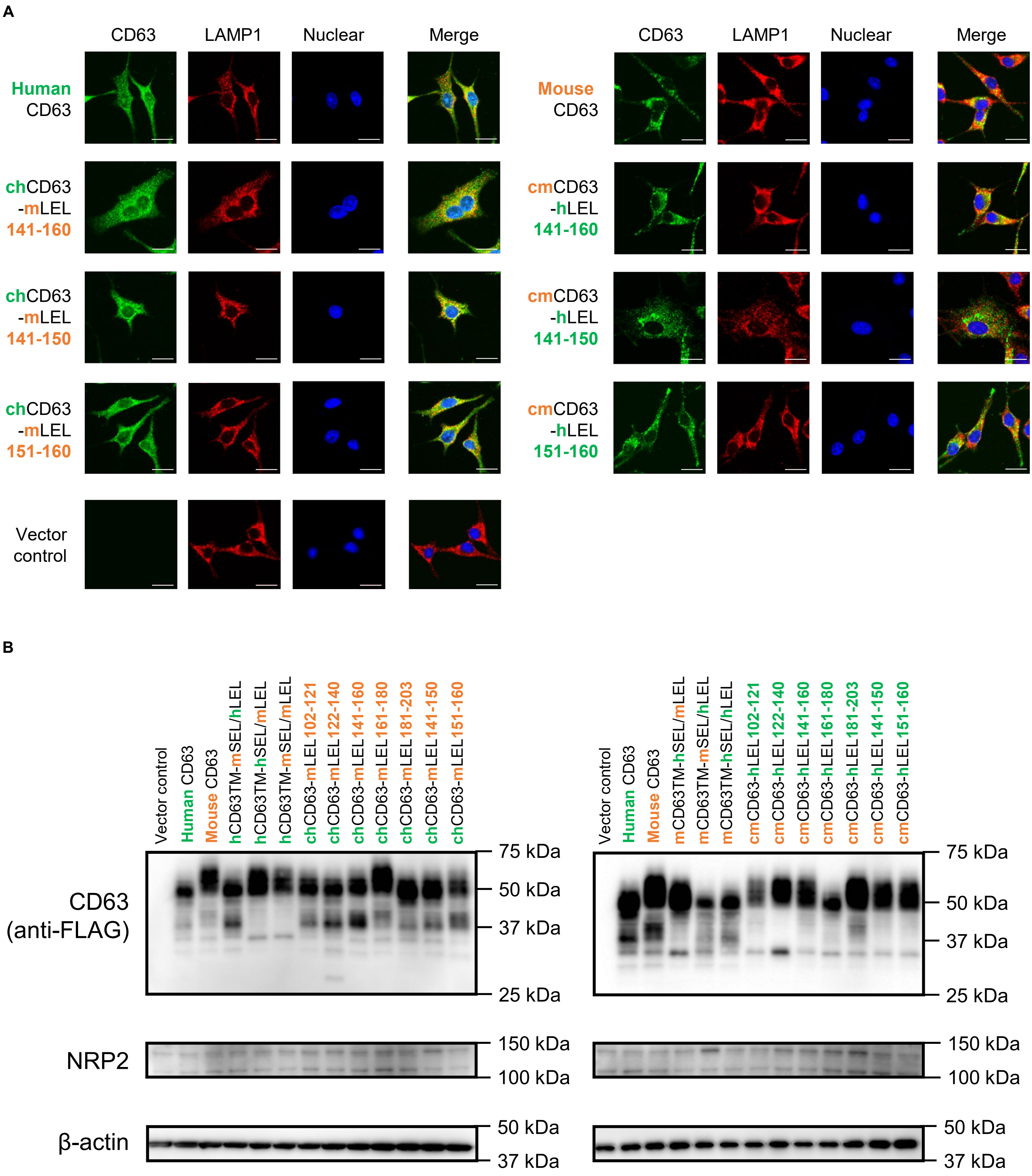

A
CD63
LAMP1
Nuclear
Merge
Human CD63
chCD63 -mLEL 141-160
chCD63 -mLEL 141-150
chCD63 -mLEL 151-160
Vector control
Mouse CD63
cmCD63 -hLEL 141-160
cmCD63 -hLEL 141-150
cmCD63 -hLEL 151-160
B
Vector control
Human CD63
Mouse CD63
hCD63TM-mSEL/hLEL
hCD63TM-hSEL/mLEL
hCD63TM-mSEL/mLEL
chCD63-mLEL102-121
chCD63-mLEL122-140
chCD63-mLEL141-160
chCD63-mLEL161-180
chCD63-mLEL181-203
chCD63-mLEL141-150
chCD63-mLEL151-160
mCD63TM-hSEL/mLEL
mCD63TM-mSEL/hLEL
mCD63TM-hSEL/hLEL
cmCD63-hLEL102-121
cmCD63-hLEL122-140
cmCD63-hLEL141-160
cmCD63-hLEL161-180
cmCD63-hLEL181-203
cmCD63-hLEL141-150
cmCD63-hLEL151-160
CD63 (anti-FLAG)
NRP2
β-actin
75 kDa
50 kDa
37 kDa
25 kDa
150 kDa
100 kDa

Figure S4

Figure S4. Expression of exogenous human and mouse CD63s and their mutants having the chimeric LEL regions in BHK cells, Related to Figure 4.

(A) The intracellular localization of CD63 in BHK cells was analyzed by confocal microscopy as described in Materials and Methods. The scale bars represent 20 μm. (B) Each cell lysate was separated in SDS-PAGE followed by western blotting as described in Materials and Methods.
