# Supplementary material for: Molecular Mechanisms Underlying the Cellular Entry and Host Range Restriction of Lujo Virus
Source: mBio. 2022 Feb 15;13(1):e03060-21. doi: 10.1128/mbio.03060-21 (PMC8844913; doi:10.1128/mbio.03060-21)
Supplement: FIG S5 [file mbio.03060-21-sf005.pdf]

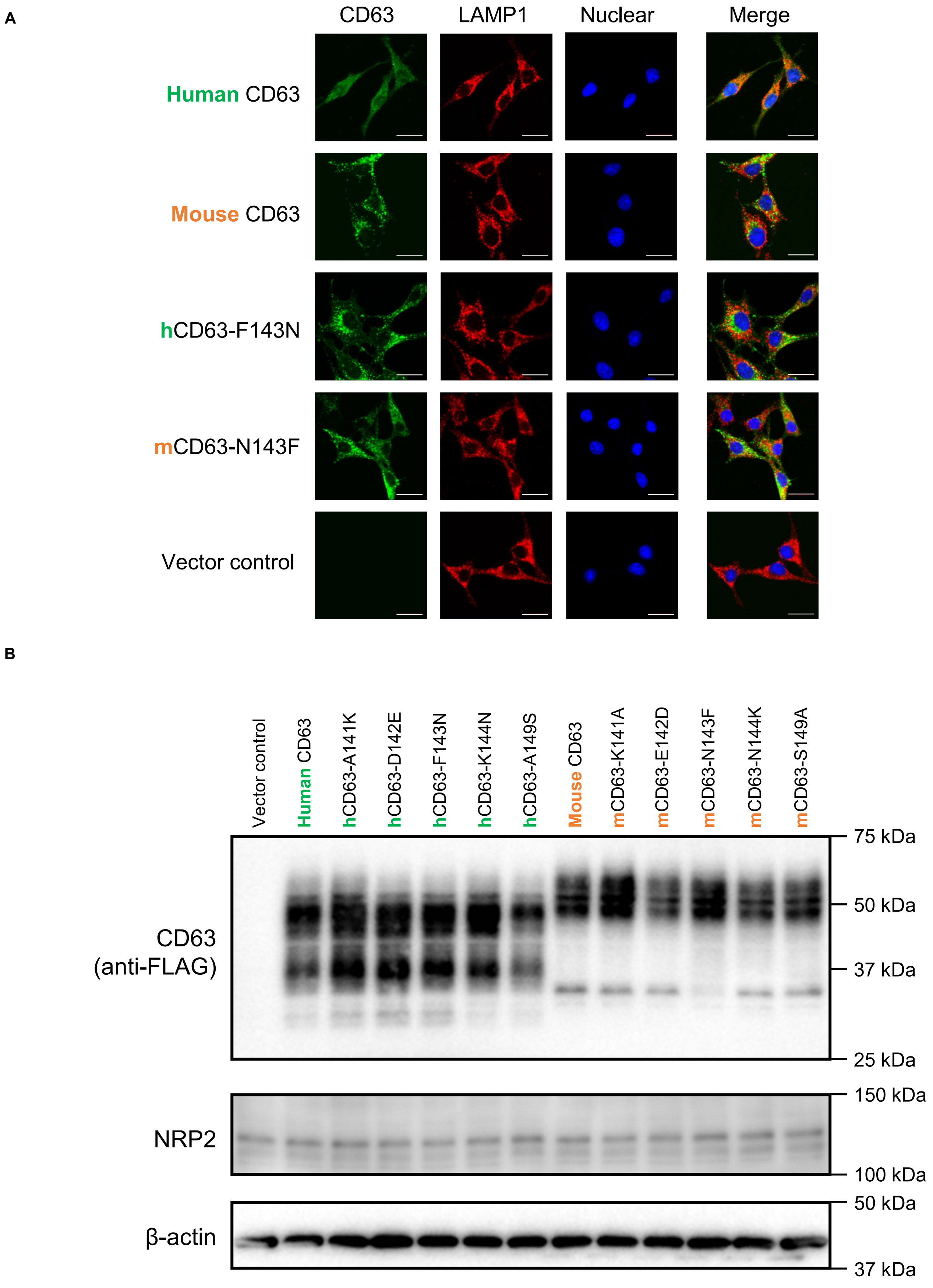
A
CD63
LAMP1
Nuclear
Merge
Human CD63
Mouse CD63
hCD63-F143N
mCD63-N143F
Vector control
B
Vector control
Human CD63
hCD63-A141K
hCD63-D142E
hCD63-F143N
hCD63-K144N
hCD63-A149S
Mouse CD63
mCD63-K141A
mCD63-E142D
mCD63-N143F
mCD63-N144K
mCD63-S149A
CD63
(anti-FLAG)
75 kDa
50 kDa
37 kDa
25 kDa
NRP2
150 kDa
100 kDa
β-actin
50 kDa
37 kDa

Figure S5

Figure S5. Expression of exogenous human and mouse CD63s and their mutants with single amino acid substitutions in BHK cells, Related to Figure 5.

(A) The intracellular localization of CD63 in BHK cells was analyzed by confocal microscopy as described in Materials and Methods. The scale bars represent 20 μm. (B) Each cell lysate was separated in SDS-PAGE followed by western blotting as described in Materials and Methods.
