# Supplementary material for: Molecular Mechanisms Underlying the Cellular Entry and Host Range Restriction of Lujo Virus
Source: mBio. 2022 Feb 15;13(1):e03060-21. doi: 10.1128/mbio.03060-21 (PMC8844913; doi:10.1128/mbio.03060-21)
Supplement: FIG S6 [file mbio.03060-21-sf006.pdf]

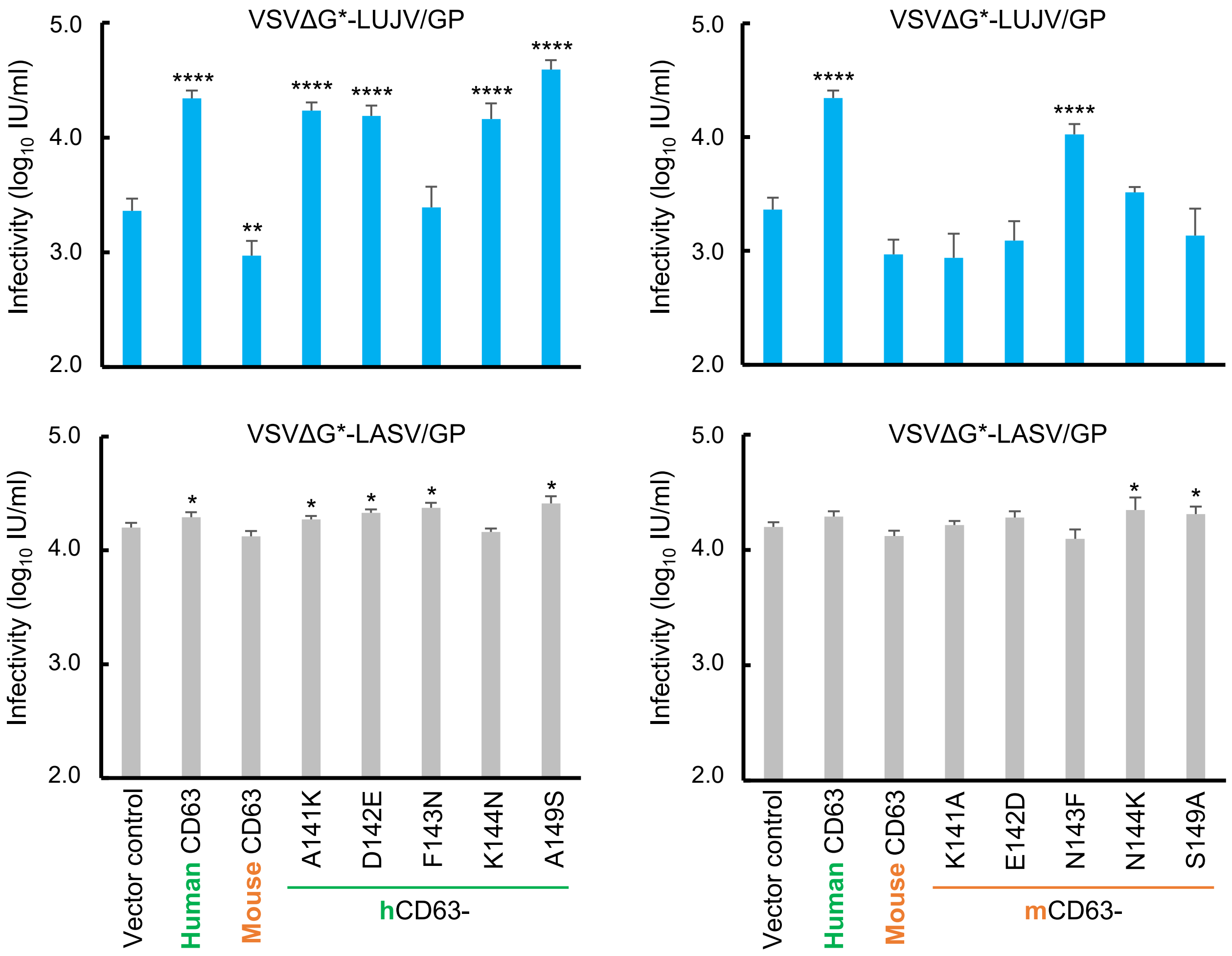

Figure S6. Importance of the amino acid residues at position 143 of human CD63 for VSVΔG*-LUJV/GP entry in NIH3T3 cells, Related to Figure 5.

The infectious units (IU) of VSVΔG*-LUJV/GP and VSVΔG*-LASV/GP in NIH3T3 cells expressing wildtype human, mouse, or mutant CD63s are shown. Each experiment was conducted three times, and averages and standard deviations are shown. Significant differences compared to the cells transduced with the vector control (Vector control) are shown (*P < 0.05, **P < 0.01, ***P < 0.001, ****P < 0.0001)
