# Supplementary material for: Molecular Mechanisms Underlying the Cellular Entry and Host Range Restriction of Lujo Virus
Source: mBio. 2022 Feb 15;13(1):e03060-21. doi: 10.1128/mbio.03060-21 (PMC8844913; doi:10.1128/mbio.03060-21)
Supplement: FIG S7 [file mbio.03060-21-sf007.pdf]

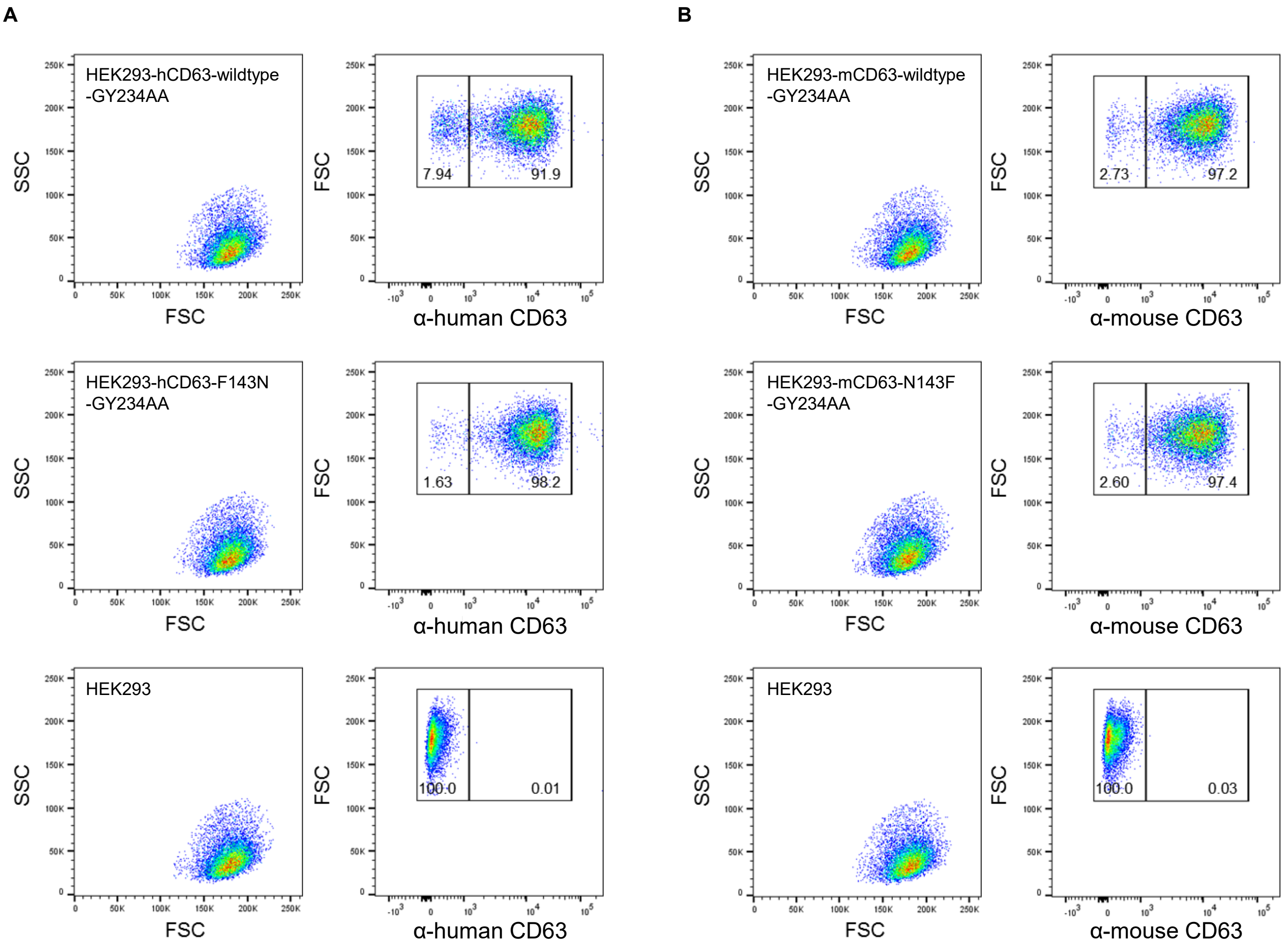


**Figure S7. Cell surface localization of exogenous CD63 in HEK293 cells shown in Figure 6.** Surface localization of the exogenous human CD63 mutants (A) and mouse CD63 mutants (B) were confirmed by flow cytometric analyses. For these analyses, mouse anti-human CD63 (A) and rat anti-mouse CD63 monoclonal antibodies (B) were used as primary antibodies. Primary antibody binding was detected with goat Alexa Fluor 488-conjugated anti-mouse IgG antibody and goat Alexa Fluor 488-conjugated anti-rat IgG antibodies. After washing, the cells were analyzed employing a FACS Conto flow cytometer (BD Biosicences) and FlowJo software (Tree Star).

Figure S7
