# Supplementary material for: Molecular Mechanisms Underlying the Cellular Entry and Host Range Restriction of Lujo Virus
Source: mBio. 2022 Feb 15;13(1):e03060-21. doi: 10.1128/mbio.03060-21 (PMC8844913; doi:10.1128/mbio.03060-21)
Supplement: TABLE S1 [file mbio.03060-21-st001.pdf]

Table S1. Comparison of amino acid sequences among animals, Related to Figure 7

| Class | Order | Common name | Species name | Accession Number | Amino acid residues at positions 140-152 (human CD63 numbering) | | | | | | | | | | | | |
|---|---|---|---|---|---|---|---|---|---|---|---|---|---|---|---|---|---|
| | | | | | 140 | 141 | 142 | 143 | 144 | 145 | 146 | 147 | 148 | 149 | 150 | 151 | 152 |
| Mammalia | Primates | Human | Homo sapiens | (NP_001771.1) | Q | A | D | F | K | C | C | G | A | A | N | Y | T |
| Mammalia | Primates | Rhesus monkey | Macaca mulatta | (NP_001253224.1) | Q | A | D | F | K | C | C | G | A | A | N | Y | T |
| Mammalia | Primates | White-tufted-ear marmoset | Callithrix jacchus | (XP_002752630.1) | Q | T | D | F | K | C | C | G | A | A | N | Y | T |
| Mammalia | Primates | Small-eared galago | Otolemur garnettii | (XP_003790548.1) | Q | E | E | F | K | C | C | G | A | A | N | Y | T |
| Mammalia | Primates | Pygmy chimpanzee | Pan paniscus | (XP_003807589.2) | Q | A | D | F | K | C | C | G | A | A | N | Y | T |
| Mammalia | Primates | Olive baboon | Papio anubis | (XP_003906585.1) | Q | A | D | F | K | C | C | G | A | A | N | Y | T |
| Mammalia | Primates | Western gorilla | Gorilla gorilla gorilla | (XP_004053356.1) | Q | A | D | F | K | C | C | G | A | A | N | Y | T |
| Mammalia | Primates | Crab-eating macaque | Macaca fascicularis | (XP_005571187.1) | Q | A | D | F | K | C | C | G | A | A | N | Y | T |
| Mammalia | Primates | African green monkey | Chlorocebus sabaeus | (XP_008001759.1) | Q | A | D | F | K | C | C | G | A | A | N | Y | T |
| Mammalia | Primates | Philippine tarsier | Carlito syrichta | (XP_008062627.1) | Q | Q | E | F | N | C | C | G | A | A | N | Y | T |
| Mammalia | Primates | Sumatran orangutan | Pongo abelii | (XP_009246133.1) | Q | A | D | F | K | C | C | G | A | A | N | Y | T |
| Mammalia | Primates | Golden snub-nosed monkey | Rhinopithecus roxellana | (XP_010376148.1) | Q | A | D | F | K | C | C | G | A | A | N | Y | T |
| Mammalia | Primates | Pig-tailed macaque | Macaca nemestrina | (XP_011726969.1) | Q | A | D | F | K | C | C | G | A | A | N | Y | T |
| Mammalia | Primates | Drill | Mandrillus leucophaeus | (XP_011825466.1) | Q | A | D | F | K | C | C | G | A | A | N | Y | T |
| Mammalia | Primates | Sooty mangabey | Cercocebus atys | (XP_011918931.1) | Q | A | D | F | K | C | C | G | A | A | N | Y | T |
| Mammalia | Primates | Mas night monkey | Aotus nancymaae | (XP_012305815.1) | Q | A | D | F | K | C | C | G | A | A | N | Y | T |
| Mammalia | Primates | Coquerels sifaka | Propithecus coquereli | (XP_012508294.1) | Q | E | D | F | K | C | C | G | A | A | N | Y | T |
| Mammalia | Primates | Gray mouse lemur | Microcebus murinus | (XP_012608451.1) | Q | Q | N | F | N | C | C | G | A | A | N | Y | T |
| Mammalia | Primates | Panamanian white-faced capuchin | Cebus imitator | (XP_017374914.1) | Q | K | D | F | K | C | C | G | A | A | N | Y | T |
| Mammalia | Primates | Black snub-nosed monkey | Rhinopithecus bieti | (XP_017708783.1) | Q | A | D | F | K | C | C | G | A | A | N | Y | T |
| Mammalia | Primates | Ugandan red Colobus | Piliocolobus tephrosceles | (XP_023066625.1) | Q | A | D | F | K | C | C | G | A | A | N | Y | T |
| Mammalia | Primates | Gelada | Theropithecus gelada | (XP_025259469.1) | Q | A | D | F | K | C | C | G | A | A | N | Y | T |
| Mammalia | Primates | Northern white-cheeked gibbon | Nomascus leucogenys | (XP_030678351.1) | Q | A | D | F | K | C | C | G | A | A | N | Y | T |
| Mammalia | Primates | Silvery gibbon | Hylobates moloch | (XP_032029358.1) | Q | A | D | F | K | C | C | G | A | A | N | Y | T |
| Mammalia | Primates | Tufted capuchin | Sapajus apella | (XP_032147439.1) | Q | T | D | F | K | C | C | G | A | A | N | Y | T |
| Mammalia | Primates | Francoiss langur | Trachypithecus francoisi | (XP_033077660.1) | Q | A | D | F | K | C | C | G | A | A | N | Y | T |
| Mammalia | Primates | Bolivian squirrel monkey | Saimiri boliviensis boliviensis | (XP_039327867.1) | Q | A | E | F | E | C | C | G | A | A | N | Y | T |
| Mammalia | Primates | Chimpanzee | Pan troglodytes | (XP_509121.1) | Q | A | D | F | K | C | C | G | A | A | N | Y | T |
| Mammalia | Rodentia | Mouse | Mus musculus | (NP_031679.1) | Q | K | E | N | N | C | C | G | A | S | N | Y | T |
| Mammalia | Rodentia | African grass rat | Arvicanthis niloticus | (XP_034343158.1) | Q | K | E | N | N | C | C | G | A | S | N | Y | T |
| Mammalia | Rodentia | Norway rat | Rattus norvegicus | (XP_008763246.1) | Q | K | E | N | K | C | C | G | A | S | N | Y | T |
| Mammalia | Rodentia | Ryukyu mouse | Mus caroli | (XP_021030049.1) | Q | K | E | N | N | C | C | G | A | S | N | Y | T |
| Mammalia | Rodentia | Shrew mouse | Mus pahari | (XP_021060674.1) | Q | K | E | N | K | C | C | G | A | S | N | Y | T |
| Mammalia | Rodentia | African woodland thicket rat | Grammomys surdaster | (XP_028642514.1) | Q | K | E | N | K | C | C | G | A | S | N | Y | T |
| Mammalia | Rodentia | Southern multimammate mouse | Mastomys coucha | (XP_031205813.1) | Q | K | D | N | K | C | C | G | A | S | N | Y | T |
| Mammalia | Rodentia | Black rat | Rattus rattus | (XP_032765219.1) | Q | K | E | N | K | C | C | G | A | S | N | Y | T |
| Mammalia | Rodentia | American beaver | Castor canadensis | (XP_020034716.1) | Q | E | D | F | K | C | C | G | A | G | N | Y | S |
| Mammalia | Rodentia | Guinea pig | Cavia porcellus | (XP_003476257.1) | Q | T | K | F | H | C | C | G | A | A | N | Y | T |
| Mammalia | Rodentia | Chinese hamster | Cricetulus griseus | (XP_003506255.1) | Q | K | E | F | E | C | C | G | A | N | N | Y | T |
| Mammalia | Rodentia | American pika | Ochotona princeps | (XP_004589696.1) | Q | E | D | Y | K | C | C | G | A | A | N | Y | T |
| Mammalia | Rodentia | Degu | Octodon degus | (XP_004647156.1) | Q | K | K | F | E | C | C | G | A | A | N | Y | S |
| Mammalia | Rodentia | Lesser Egyptian jerboa | Jaculus jaculus | (XP_004650010.1) | Q | K | D | F | M | C | C | G | A | A | N | Y | T |
| Mammalia | Rodentia | Naked mole-rat | Heterocephalus glaber | (XP_004861432.1) | Q | T | K | F | E | C | C | G | A | A | N | F | T |
| Mammalia | Rodentia | Syrian hamster | Mesocricetus auratus | (XP_005079748.1) | Q | K | E | F | K | C | C | G | A | N | N | Y | T |
| Mammalia | Rodentia | Thirteen-lined ground squirrel | Ictidomys tridecemlineatus | (XP_005335651.1) | Q | K | E | F | N | C | C | G | A | T | N | Y | T |
| Mammalia | Rodentia | Prairie vole | Microtus ochrogaster | (XP_005371209.1) | Q | Q | R | F | N | C | C | G | A | N | N | Y | T |
| Mammalia | Rodentia | Prairie deer mouse | Peromyscus maniculatus bairdii | (XP_006987370.1) | Q | K | E | F | G | C | C | G | A | N | N | Y | T |
| Mammalia | Rodentia | Upper Galilee mountains blind mole rat | Nannospalax galili | (XP_008837215.1) | Q | K | K | F | Q | C | C | G | A | T | N | Y | T |
| Mammalia | Rodentia | Damara mole-rat | Fukomys damarensis | (XP_010643135.1) | Q | T | E | F | E | C | C | G | A | A | N | F | T |
| Mammalia | Rodentia | Ords kangaroo rat | Dipodomys ordii | (XP_012878611.1) | Q | K | R | F | S | C | C | G | A | S | N | Y | T |
| Mammalia | Rodentia | Chinchilla | Chinchilla lanigera | (XP_013376423.1) | Q | T | K | F | E | C | C | G | A | A | N | Y | S |
| Mammalia | Rodentia | Alpine marmot | Marmota marmota marmota | (XP_015338751.1) | Q | E | E | F | N | C | C | G | A | A | N | Y | T |
| Mammalia | Rodentia | Mongolian gerbil | Meriones unguiculatus | (XP_021515820.1) | Q | K | K | F | E | C | C | G | A | T | N | Y | T |
| Mammalia | Rodentia | Arctic ground squirrel | Urocitellus parryii | (XP_026254149.1) | Q | E | E | F | N | C | C | G | A | A | N | Y | T |
| Mammalia | Rodentia | Yellow-bellied marmot | Marmota flaviventris | (XP_027784511.1) | Q | E | E | F | N | C | C | G | A | A | N | Y | T |
| Mammalia | Rodentia | White-footed mouse | Peromyscus leucopus | (XP_028711327.1) | Q | K | E | F | G | C | C | G | A | N | N | Y | T |
| Mammalia | Rodentia | Southern grasshopper mouse | Onychomys torridus | (XP_036025748.1) | Q | K | E | F | G | C | C | G | A | N | N | Y | T |
| Mammalia | Afrosoricida | Cape golden mole | Chrysochloris asiatica | (XP_006859456.1) | Q | Q | N | F | K | C | C | G | A | F | N | Y | T |
| Mammalia | Afrosoricida | Lesser hedgehog tenrec | Echinops telfairi | (XP_004700629.1) | Q | E | D | F | K | C | C | G | A | T | N | Y | T |
| Mammalia | Artiodactyla | Minke whale | Balaenoptera acutorostrata scammoni | (XP_007179597.1) | Q | E | D | F | K | C | C | G | A | A | N | Y | T |
| Mammalia | Artiodactyla | Blue whale | Balaenoptera musculus | (XP_036723207.1) | Q | E | D | F | K | C | C | G | A | A | N | Y | T |
| Mammalia | Artiodactyla | Bison | Bison bison bison | (XP_010837479.1) | Q | K | D | F | E | C | C | G | A | A | N | Y | T |
| Mammalia | Artiodactyla | Zebu cattle | Bos indicus | (XP_019816657.1) | Q | K | D | F | E | C | C | G | A | A | N | Y | T |
| Mammalia | Artiodactyla | Hybrid cattle | Bos indicus x Bos taurus | (XP_027397946.1) | Q | K | D | F | E | C | C | G | A | A | N | Y | T |
| Mammalia | Artiodactyla | Yak | Bos mutus | (XP_005905938.1) | Q | K | D | F | E | C | C | G | A | A | N | Y | T |
| Mammalia | Artiodactyla | Cattle | Bos taurus | (NP_991372.1) | Q | K | D | F | E | C | C | G | A | A | N | Y | T |
| Mammalia | Artiodactyla | Water buffalo | Bubalus bubalis | (XP_006075148.1) | Q | K | D | F | E | C | C | G | A | A | N | Y | T |
| Mammalia | Artiodactyla | Bactrian camel | Camelus bactrianus | (XP_010962205.1) | Q | E | K | F | E | C | C | G | A | A | N | Y | T |
| Mammalia | Artiodactyla | Arabian camel | Camelus dromedarius | (XP_031319545.1) | Q | E | K | F | E | C | C | G | A | A | N | Y | T |
| Mammalia | Artiodactyla | Wild Bactrian camel | Camelus ferus | (XP_032349017.1) | Q | E | K | F | E | C | C | G | A | A | N | Y | T |
| Mammalia | Artiodactyla | Goat | Capra hircus | (XP_017903608.1) | Q | K | D | F | K | C | C | G | A | A | N | Y | T |
| Mammalia | Artiodactyla | Beluga whale | Delphinapterus leucas | (XP_022427636.1) | Q | E | D | F | K | C | C | G | A | A | N | Y | T |
| Mammalia | Artiodactyla | Long-finned pilot whale | Globicephala melas | (XP_030722585.1) | Q | E | D | F | K | C | C | G | A | A | N | Y | T |
| Mammalia | Artiodactyla | Pacific white-sided dolphin | Lagenorhynchus obliquidens | (XP_026962369.1) | Q | E | E | F | K | C | C | G | A | A | N | Y | T |
| Mammalia | Artiodactyla | Yangtze River dolphin | Lipotes vexillifer | (XP_007467827.1) | Q | E | D | F | K | C | C | G | A | A | N | Y | T |
| Mammalia | Artiodactyla | Narwhal | Monodon monoceros | (XP_029059971.1) | Q | E | D | F | K | C | C | G | A | A | N | Y | T |
| Mammalia | Artiodactyla | Yangtze finless porpoise | Neophocaena asiaeorientalis asiaeorientalis | (XP_024592540.1) | Q | E | D | F | K | C | C | G | A | A | N | Y | T |
| Mammalia | Artiodactyla | White-tailed deer | Odocoileus virginianus texanus | (XP_020764156.1) | Q | K | E | F | K | C | C | G | A | A | N | Y | T |
| Mammalia | Artiodactyla | Killer whale | Orcinus orca | (XP_004274244.1) | Q | E | D | F | K | C | C | G | A | A | N | Y | T |
| Mammalia | Artiodactyla | Sheep | Ovis aries | (XP_027823415.1) | Q | K | D | F | K | C | C | G | A | A | N | Y | T |
| Mammalia | Artiodactyla | Vaquita | Phocoena sinus | (XP_032502944.1) | Q | E | D | F | K | C | C | G | A | A | N | Y | T |
| Mammalia | Artiodactyla | Sperm whale | Physeter catodon | (XP_007125753.1) | Q | K | D | F | K | C | C | G | A | A | N | Y | T |
| Mammalia | Artiodactyla | Pig | Sus scrofa | (XP_005663935.1) | Q | E | D | F | K | C | C | G | A | A | N | Y | T |
| Mammalia | Artiodactyla | Common bottlenose dolphin | Tursiops truncatus | (XP_033722413.1) | Q | E | D | F | K | C | C | G | A | A | N | Y | T |
| Mammalia | Artiodactyla | Alpaca | Vicugna pacos | (XP_006218608.1) | Q | E | K | F | E | C | C | G | A | A | N | Y | T |
| Mammalia | Carnivora | Cheetah | Acinonyx jubatus | (XP_026927340.1) | Q | E | D | F | K | C | C | G | A | A | N | Y | T |
| Mammalia | Carnivora | Giant panda | Ailuropoda melanoleuca | (XP_034500044.1) | Q | E | D | F | K | C | C | G | A | A | N | Y | T |
| Mammalia | Carnivora | Northern fur seal | Callorhinus ursinus | (XP_025738584.1) | Q | E | D | F | K | C | C | G | A | A | N | Y | T |
| Mammalia | Carnivora | Dingo | Canis lupus dingo | (XP_025332882.1) | Q | E | D | F | K | C | C | G | A | A | N | Y | T |
| Mammalia | Carnivora | Dog | Canis lupus familiaris | (XP_003639366.1) | Q | E | D | F | K | C | C | G | A | A | N | Y | T |
| Mammalia | Carnivora | Sea otter | Enhydra lutris kenyoni | (XP_022378727.1) | Q | E | D | F | K | C | C | G | A | A | N | Y | T |
| Mammalia | Carnivora | Steller sea lion | Eumetopias jubatus | (XP_027966756.1) | Q | E | D | F | K | C | C | G | A | A | N | Y | T |
| Mammalia | Carnivora | Cat | Felis catus | (NP_001009855.1) | Q | E | D | F | K | C | C | G | A | A | N | Y | T |
| Mammalia | Carnivora | Gray seal | Halichoerus grypus | (XP_035942463.1) | Q | E | D | F | K | C | C | G | A | A | N | Y | T |
| Mammalia | Carnivora | Weddell seal | Leptonychotes weddellii | (XP_006728807.1) | Q | E | D | F | K | C | C | G | A | A | N | Y | T |
| Mammalia | Carnivora | Northern American river otter | Lontra canadensis | (XP_032730279.1) | Q | E | D | F | K | C | C | G | A | A | N | Y | T |
| Mammalia | Carnivora | Canada lynx | Lynx canadensis | (XP_030178705.1) | Q | E | D | F | K | C | C | G | A | A | N | Y | T |
| Mammalia | Carnivora | Southern elephant seal | Mirounga leonina | (XP_034858339.1) | Q | E | D | F | K | C | C | G | A | A | N | Y | T |
| Mammalia | Carnivora | Ermine | Mustela erminea | (XP_032204424.1) | Q | E | D | F | K | C | C | G | A | A | N | Y | T |
| Mammalia | Carnivora | Ferret | Mustela putorius furo | (XP_004773100.1) | Q | E | D | F | K | C | C | G | A | A | N | Y | T |
| Mammalia | Carnivora | Hawaiian monk seal | Neomonachus schauinslandi | (XP_021542839.1) | Q | E | D | F | K | C | C | G | A | A | N | Y | T |
| Mammalia | Carnivora | Pacific walrus | Odobenus rosmarus divergens | (XP_012418302.1) | Q | E | D | F | K | C | C | G | A | A | N | Y | T |
| Mammalia | Carnivora | Leopard | Panthera pardus | (XP_019310240.1) | Q | E | D | F | K | C | C | G | A | A | N | Y | T |
| Mammalia | Carnivora | Amur tiger | Panthera tigris altaica | (XP_007081525.1) | Q | E | D | F | K | C | C | G | A | A | N | Y | T |
| Mammalia | Carnivora | Harbor seal | Phoca vitulina | (XP_032276427.1) | Q | E | D | F | K | C | C | G | A | A | N | Y | T |
| Mammalia | Carnivora | Puma | Puma concolor | (XP_025782857.1) | Q | E | D | F | K | C | C | G | A | A | N | Y | T |
| Mammalia | Carnivora | Meerkat | Suricata suricatta | (XP_029809929.1) | Q | E | D | F | Q | C | C | G | A | A | N | Y | T |
| Mammalia | Carnivora | Grizzly bear | Ursus arctos horribilis | (XP_026356112.1) | Q | E | D | F | K | C | C | G | A | A | N | Y | T |
| Mammalia | Carnivora | Polar bear | Ursus maritimus | (XP_040493044.1) | Q | E | D | F | K | C | C | G | A | A | N | Y | T |
| Mammalia | Carnivora | Red fox | Vulpes vulpes | (XP_025857789.1) | Q | E | D | F | K | C | C | G | A | A | N | Y | T |
| Mammalia | Carnivora | California sea lion | Zalophus californianus | (XP_027451224.1) | Q | E | D | F | K | C | C | G | A | A | N | Y | T |
| Mammalia | Cingulata | Nine-banded armadillo | Dasypus novemcinctus | (XP_004466121.1) | Q | R | D | F | K | C | C | G | A | T | N | Y | S |
| Mammalia | Dasyuromorphia | Tasmanian devil | Sarcophilus harrisii | (XP_023361044.1) | Q | K | E | F | T | C | C | G | A | A | N | Y | T |
| Mammalia | Dermoptera | Sunda flying lemur | Galeopterus variegatus | (XP_008581357.1) | Q | E | H | F | N | C | C | G | A | A | N | Y | T |
| Mammalia | Diprotodontia | Koala | Phascolarctos cinereus | (XP_020845615.1) | Q | E | G | F | K | C | C | G | A | V | N | Y | T |
| Mammalia | Diprotodontia | Common brushtail | Trichosurus vulpecula | (XP_036617685.1) | Q | K | D | F | K | C | C | G | A | T | N | Y | T |
| Mammalia | Diprotodontia | Common wombat | Vombatus ursinus | (XP_027710807.1) | Q | E | G | F | K | C | C | G | A | M | N | Y | T |
| Mammalia | Eulipotyphla | Star-nosed mole | Condylura cristata | (XP_012587744.1) | Q | Q | E | F | Q | C | C | G | A | V | N | Y | T |
| Mammalia | Eulipotyphla | Western European hedgehog | Erinaceus europaeus | (XP_007533427.1) | Q | K | E | F | K | C | C | G | A | H | N | Y | T |
| Mammalia | Eulipotyphla | European shrew | Sorex araneus | (XP_004601979.1) | Q | E | R | F | N | C | C | G | A | D | N | Y | T |
| Mammalia | Eulipotyphla | Iberian mole | Talpa occidentalis | (XP_037369213.1) | Q | Q | K | F | Q | C | C | G | A | D | N | Y | T |

| | | | | | | | | | | | | | | | | | |
|---|---|---|---|---|---|---|---|---|---|---|---|---|---|---|---|---|---|
| *Mammalia* | *Lagomorpha* | Rabbit | *Oryctolagus cuniculus* | (NP_001075668.1) | Q | **K** | **D** | F | **T** | C | C | G | A | **A** | N | Y | T |
| *Mammalia* | *Macroscelidea* | Cape elephant shrew | *Elephantulus edwardii* | (XP_006897717.1) | Q | **Q** | **E** | F | **K** | C | C | G | A | **A** | N | Y | T |
| *Mammalia* | *Monotremata* | Platypus | *Ornithorhynchus anatinus* | (XP_028928536.1) | Q | **K** | **D** | F | **K** | C | C | G | A | **H** | N | Y | T |
| *Mammalia* | *Perissodactyla* | Southern white rhinoceros | *Ceratotherium simum simum* | (XP_004429265.1) | Q | **E** | **E** | F | **K** | C | C | G | A | **V** | N | Y | T |
| *Mammalia* | *Perissodactyla* | Asinus | *Equus asinus* | (XP_014712740.1) | Q | **E** | **K** | F | **H** | C | C | G | A | **T** | N | Y | T |
| *Mammalia* | *Perissodactyla* | Horse | *Equus caballus* | (XP_023499518.1) | Q | **E** | **K** | F | **H** | C | C | G | A | **T** | N | Y | T |
| *Mammalia* | *Perissodactyla* | Przewalskis horse | *Equus przewalskii* | (XP_008532416.1) | Q | **E** | **K** | F | **H** | C | C | G | A | **T** | N | Y | T |
| *Mammalia* | *Pholidota* | Malayan pangolin | *Manis javanica* | (XP_036864822.1) | Q | **K** | **D** | F | **K** | C | C | G | A | **A** | N | Y | T |
| *Mammalia* | *Pholidota* | Chinese pangolin | *Manis pentadactyla* | (XP_036774242.1) | Q | **K** | **D** | F | **K** | C | C | G | A | **A** | N | Y | T |
| *Mammalia* | *Proboscidea* | African savanna elephant | *Loxodonta africana* | (XP_003405156.1) | Q | **Q** | **D** | F | **K** | C | C | G | A | **N** | N | Y | T |
| *Mammalia* | *Scandentia* | Chinese tree shrew | *Tupaia chinensis* | (XP_006165626.1) | Q | **N** | **Q** | S | **H** | C | C | G | A | **T** | N | Y | T |
| *Mammalia* | *Sirenia* | Florida manatee | *Trichechus manatus latirostris* | (XP_012412029.1) | Q | **R** | **D** | F | **K** | C | C | G | A | **D** | N | Y | T |
| *Mammalia* | *Tubulidentata* | Orycteropus afer | *Orycteropus afer afer* | (XP_007953272.1) | Q | **Q** | **E** | F | **K** | C | C | G | A | **A** | N | Y | T |
| *Mammalia* | *Chiroptera* | Large flying fox | *Pteropus vampyrus* | (XP_011374032.1) | Q | **E** | **N** | F | **K** | C | C | G | A | **A** | N | Y | T |
| *Mammalia* | *Chiroptera* | Jamaican fruit-eating bat | *Artibeus jamaicensis* | (XP_037000026.1) | Q | **E** | **E** | F | **N** | C | C | G | A | **A** | N | Y | T |
| *Mammalia* | *Chiroptera* | Common vampire bat | *Desmodus rotundus* | (XP_024432083.1) | Q | **E** | **N** | F | **K** | C | C | G | A | **A** | N | Y | T |
| *Mammalia* | *Chiroptera* | Big brown bat | *Eptesicus fuscus* | (XP_008146815.1) | Q | **K** | **E** | F | **K** | C | C | G | A | **A** | N | Y | T |
| *Mammalia* | *Chiroptera* | Great roundleaf bat | *Hipposideros armiger* | (XP_019524679.1) | Q | **K** | **G** | F | **N** | C | C | G | A | **A** | N | Y | T |
| *Mammalia* | *Chiroptera* | Natal long-fingered bat | *Miniopterus natalensis* | (XP_016069874.1) | Q | **K** | **K** | F | **R** | C | C | G | A | **A** | N | Y | T |
| *Mammalia* | *Chiroptera* | Pallass mastiff bat | *Molossus molossus* | (XP_036107955.1) | Q | **K** | **N** | F | **R** | C | C | G | A | **A** | N | Y | T |
| *Mammalia* | *Chiroptera* | Brandts bat | *Myotis brandtii* | (XP_014403621.1) | Q | **K** | **K** | F | **K** | C | C | G | A | **A** | N | Y | T |
| *Mammalia* | *Chiroptera* | David's myotis | *Myotis davidii* | (XP_006777885.2) | Q | **K** | **K** | F | **N** | C | C | G | A | **A** | N | Y | T |
| *Mammalia* | *Chiroptera* | Little brown bat | *Myotis lucifugus* | (XP_014315480.1) | Q | **K** | **K** | F | **K** | C | C | G | A | **A** | N | Y | T |
| *Mammalia* | *Chiroptera* | Greater mouse-eared ba | *Myotis myotis* | (XP_036156549.1) | Q | **K** | **K** | F | **K** | C | C | G | A | **A** | N | Y | T |
| *Mammalia* | *Chiroptera* | Pale spear-nosed bat | *Phyllostomus discolor* | (XP_028388955.1) | Q | **E** | **N** | F | **K** | C | C | G | A | **A** | N | Y | T |
| *Mammalia* | *Chiroptera* | Kuhls pipistrelle | *Pipistrellus kuhlii* | (XP_036314246.1) | Q | **T** | **Q** | F | **S** | C | C | G | A | **D** | N | Y | T |
| *Mammalia* | *Chiroptera* | Black flying fox | *Pteropus alecto* | (XP_006908925.1) | Q | **E** | **N** | F | **K** | C | C | G | A | **A** | N | Y | T |
| *Mammalia* | *Chiroptera* | Greater horseshoe bat | *Rhinolophus ferrumequinum* | (XP_032974550.1) | Q | **E** | **N** | F | **E** | C | C | G | A | **A** | N | Y | T |
| *Mammalia* | *Chiroptera* | Egyptian fruit bat | *Rousettus aegyptiacus* | (XP_016021290.2) | Q | **E** | **N** | F | **K** | C | C | G | A | **A** | N | Y | T |
| *Mammalia* | *Chiroptera* | Highland yellow-shouldered bat | *Sturnira hondurensis* | (XP_036885903.1) | Q | **E** | **K** | F | **K** | C | C | G | A | **A** | N | Y | T |
| *Aves* | *Accipitriformes* | Bald eagle | *Haliaeetus leucocephalus* | (XP_010563405.1) | Q | S | **D** | F | G | C | C | G | A | N | N | Y | T |
| *Aves* | *Accipitriformes* | Golden eagle | *Aquila chrysaetos chrysaetos* | (XP_029892816.1) | Q | R | **D** | F | S | C | C | G | A | N | N | Y | T |
| *Aves* | *Anseriformes* | Tufted duck | *Aythya fuligula* | (XP_032060667.1) | Q | E | **D** | F | H | C | C | G | A | **S** | N | Y | T |
| *Aves* | *Anseriformes* | Black swan | *Cygnus atratus* | (XP_035427514.1) | Q | E | **D** | F | H | C | C | G | A | **S** | N | Y | T |
| *Aves* | *Columbiformes* | Rock pigeon | *Columba livia* | (XP_021138894.1) | Q | R | **D** | F | Q | C | C | G | A | N | N | Y | T |
| *Aves* | *Galliformes* | Turkey | *Meleagris gallopavo* | (XP_010725346.1) | Q | E | **D** | F | H | C | C | G | S | N | N | Y | T |
| *Aves* | *Galliformes* | Chicken | *Gallus gallus* | (XP_015128066.1) | Q | E | **D** | F | H | C | C | G | A | N | N | Y | T |
| *Aves* | *Galliformes* | Ring-necked pheasant | *Phasianus colchicus* | (XP_031459082.1) | Q | E | **D** | F | H | C | C | G | S | N | N | Y | T |
| *Aves* | *Passeriformes* | Tibetan ground-tit | *Pseudopodoces humilis* | (XP_014117933.1) | Q | Q | **E** | F | S | C | C | G | V | Q | N | Y | T |
| *Aves* | *Passeriformes* | Common starling | *Sturnus vulgaris* | (XP_014748853.1) | Q | Q | **E** | F | S | C | C | G | V | N | N | Y | T |
| *Aves* | *Passeriformes* | Blue-crowned manakin | *Lepidothrix coronata* | (XP_017694170.1) | Q | R | **D** | F | K | C | C | G | A | N | N | Y | T |
| *Aves* | *Passeriformes* | White-ruffed manakin | *Corapipo altera* | (XP_027525100.1) | Q | R | K | F | K | C | C | G | A | N | N | Y | T |
| *Aves* | *Passeriformes* | Saffron-crested tyrant-manakin | *Neopelma chrysocephalum* | (XP_027561344.1) | Q | R | **E** | F | K | C | C | G | A | N | N | Y | T |
| *Aves* | *Passeriformes* | Willow flycatcher | *Empidonax traillii* | (XP_027763110.1) | Q | R | N | F | **N** | C | C | G | A | I | N | Y | T |
| *Aves* | *Passeriformes* | Small tree finch | *Camarhynchus parvulus* | (XP_030823157.1) | Q | Q | **E** | F | S | C | C | G | V | N | N | Y | T |
| *Aves* | *Passeriformes* | Bengalese finch | *Lonchura striata domestica* | (XP_031363075.1) | Q | R | **E** | L | S | C | C | G | V | N | N | Y | T |
| *Aves* | *Passeriformes* | Lance-tailed manakin | *Chiroxiphia lanceolata* | (XP_032569541.1) | Q | R | K | F | K | C | C | G | A | N | N | Y | T |
| *Aves* | *Passeriformes* | Swainson's thrush | *Catharus ustulatus* | (XP_032939294.1) | Q | Q | **E** | F | S | C | C | G | V | N | N | F | T |
| *Aves* | *Psittaciformes* | Kakapo | *Strigops habroptila* | (XP_030366235.1) | Q | R | **D** | F | L | C | C | G | A | N | N | Y | T |
| *Aves* | *Psittaciformes* | Budgerigar | *Melopsittacus undulatus* | (XP_033927364.1) | Q | R | R | F | F | C | C | G | A | N | N | Y | T |
| *Aves* | *Tinamiformes* | Chilean tinamou | *Nothoprocta perdicaria* | (XP_025903162.1) | Q | E | **E** | F | H | C | C | G | I | N | N | Y | T |
| *Reptilia* | *Crocodilia* | Chinese alligator | *Alligator sinensis* | (XP_014380967.1) | Q | **K** | Q | Y | M | C | C | G | A | N | N | Y | T |
| *Reptilia* | *Crocodilia* | American alligator | *Alligator mississippiensis* | (XP_014451999.1) | Q | **K** | Q | Y | M | C | C | G | A | N | N | Y | T |
| *Reptilia* | *Squamata* | Green anole | *Anolis carolinensis* | (XP_003223232.1) | Q | R | T | Y | R | C | C | G | A | **S** | N | Y | S |
| *Reptilia* | *Squamata* | Burmese python | *Python bivittatus* | (XP_007435488.1) | Q | **K** | **E** | Y | S | C | C | G | A | L | N | Y | T |
| *Reptilia* | *Squamata* | Common garter snake | *Thamnophis sirtalis* | (XP_013916031.1) | Q | **K** | K | F | S | C | C | G | I | N | R | Y | S |
| *Reptilia* | *Squamata* | Schlegel's Japanese gecko | *Gekko japonicus* | (XP_015280177.1) | Q | T | **D** | F | S | C | C | G | A | **A** | N | Y | T |
| *Reptilia* | *Squamata* | Venomous pit viper | *Protobothrops mucrosquamatus* | (XP_015679685.1) | Q | **K** | K | Y | S | C | C | G | V | T | N | Y | T |
| *Reptilia* | *Squamata* | Japanese quail | *Coturnix japonica* | (XP_015742409.1) | Q | E | **D** | F | H | C | C | G | A | N | N | Y | T |
| *Reptilia* | *Squamata* | Central bearded dragon | *Pogona vitticeps* | (XP_020639030.1) | Q | E | K | Y | **N** | C | C | G | A | F | N | Y | T |
| *Reptilia* | *Squamata* | Mainland tiger snake | *Notechis scutatus* | (XP_026536620.1) | Q | **K** | K | Y | S | C | C | G | V | T | S | Y | T |
| *Reptilia* | *Squamata* | Eastern brown snake | *Pseudonaja textilis* | (XP_026566939.1) | Q | **K** | K | Y | S | C | C | G | V | **A** | S | Y | T |
| *Reptilia* | *Squamata* | Common wall lizard | *Podarcis muralis* | (XP_028567639.1) | Q | Q | K | Y | S | C | C | G | A | **S** | N | Y | T |
| *Reptilia* | *Squamata* | Western terrestrial garter snake | *Thamnophis elegans* | (XP_032065835.1) | Q | **K** | K | F | S | C | C | G | I | N | R | Y | S |
| *Reptilia* | *Squamata* | Sand lizard | *Lacerta agilis* | (XP_032994666.1) | Q | Q | K | Y | **N** | C | C | G | A | F | N | Y | T |
| *Reptilia* | *Squamata* | Corn snake | *Pantherophis guttatus* | (XP_034268427.1) | Q | **K** | R | Y | S | C | C | G | I | N | S | Y | T |
| *Reptilia* | *Squamata* | Common lizard | *Zootoca vivipara* | (XP_034959776.1) | Q | E | K | Y | S | C | C | G | A | F | N | Y | T |
| *Reptilia* | *Testudines* | Painted turtle | *Chrysemys picta* | (XP_005308480.1) | Q | **K** | K | Y | S | C | C | G | A | H | N | Y | T |
| *Reptilia* | *Testudines* | Three-toed box turtle | *Terrapene carolina triunguis* | (XP_026517257.1) | Q | **K** | K | Y | S | C | C | G | A | R | N | Y | T |
| *Reptilia* | *Testudines* | Goodes thornscrub tortoise | *Gopherus evgoodei* | (XP_030410680.1) | Q | **K** | T | Y | S | C | C | G | A | Q | N | Y | T |
| *Reptilia* | *Testudines* | Red-eared slider turtle | *Trachemys scripta elegans* | (XP_034648803.1) | Q | **K** | K | Y | S | C | C | G | A | H | N | Y | T |
| *Reptilia* | *Testudines* | Green sea turtle | *Chelonia mydas* | (XP_037739900.1) | Q | R | K | Y | S | C | C | G | A | N | N | Y | T |
| *Amphibia* | *Anura* | Tropical clawed frog | *Xenopus tropicalis* | (NP_001016413.1) | Q | **K** | **E** | F | Q | C | C | G | A | I | N | S | T |
| *Amphibia* | *Anura* | High Himalaya frog | *Nanorana parkeri* | (XP_018423488.1) | Q | **K** | A | F | K | C | C | G | A | N | A | S | S |
| *Amphibia* | *Gymnophiona* | Two-lined caecilian | *Rhinatrema bivittatum* | (XP_029450550.1) | Q | R | **D** | F | K | C | C | G | E | N | G | S | T |
| *Amphibia* | *Gymnophiona* | | *Microcaecilia unicolor* | (XP_030052646.1) | Q | R | S | F | **N** | C | C | G | A | **A** | N | Y | T |
| *Amphibia* | *Gymnophiona* | Gaboon caecilian | *Geotrypetes seraphini* | (XP_033793593.1) | Q | H | N | F | K | C | C | G | A | N | N | S | E |
